# The Radical SAM Heme Synthase AhbD from *Methanosarcina barkeri* Contains Two Auxiliary [4Fe-4S] Clusters

**DOI:** 10.3390/biom13081268

**Published:** 2023-08-18

**Authors:** Isabelle Fix, Lorenz Heidinger, Thorsten Friedrich, Gunhild Layer

**Affiliations:** 1 Institut für Pharmazeutische Wissenschaften, Pharmazeutische Biologie, Albert-Ludwigs-Universität Freiburg, Stefan-Meier-Str. 19, 79104 Freiburg, Germany; 2 Institut für Biochemie, Albert-Ludwigs-Universität Freiburg, Albertstr. 21, 79104 Freiburg, Germany; lorenz.heidinger@bio.chemie.uni-freiburg.de (L.H.); thorsten.friedrich@bio.chemie.uni-freiburg.de (T.F.)

**Keywords:** siroheme-dependent heme biosynthesis, Radical SAM enzymes, SPASM domain, iron–sulfur cluster

## Abstract

In archaea and sulfate-reducing bacteria, heme is synthesized via the siroheme-dependent pathway. The last step of this route is catalyzed by the Radical SAM enzyme AhbD and consists of the conversion of iron-coproporphyrin III into heme. AhbD belongs to the subfamily of Radical SAM enzymes containing a SPASM/Twitch domain carrying either one or two auxiliary iron–sulfur clusters in addition to the characteristic Radical SAM cluster. In previous studies, AhbD was reported to contain one auxiliary [4Fe-4S] cluster. In this study, the amino acid sequence motifs containing conserved cysteine residues in AhbD proteins from different archaea and sulfate-reducing bacteria were reanalyzed. Amino acid sequence alignments and computational structural models of AhbD suggested that a subset of AhbD proteins possesses the full SPASM motif and might contain two auxiliary iron–sulfur clusters (AuxI and AuxII). Therefore, the cluster content of AhbD from *Methanosarcina barkeri* was studied using enzyme variants lacking individual clusters. The purified enzymes were analyzed using UV/Visible absorption and EPR spectroscopy as well as iron/sulfide determinations showing that AhbD from *M. barkeri* contains two auxiliary [4Fe-4S] clusters. Heme synthase activity assays suggested that the AuxI cluster might be involved in binding the reaction intermediate and both clusters potentially participate in electron transfer.

## 1. Introduction

**Figure 1:**
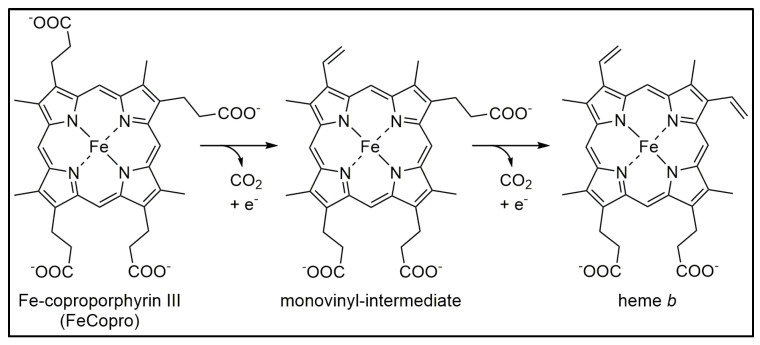
Reaction catalyzed by AhbD. The oxidative decarboxylation of the two propionate side chains at pyrrole rings A and B of FeCopro proceeds stepwise with a detectable monovinyl-intermediate. In the reaction scheme, only one of the two possible intermediates is shown.

The cyclic, iron-containing tetrapyrrole heme plays an important role in almost all organisms by fulfilling one or several different functions ranging from oxygen transport and gas sensing to electron transfer and catalysis [1]. Depending on the organism, heme can be acquired from the environment by dedicated uptake systems or it is biosynthesized by either one of three different pathways [2]. The first branch point, at which the different heme biosynthesis routes diverge, occurs at the stage of the last common precursor of all tetrapyrroles, namely uroporphyrinogen III. While eukaryotes and many Gram-negative bacteria utilize the protoporphyrin-dependent (PPD) pathway, Gram-positive bacteria synthesize heme via the coproporphyrin-dependent (CPD) route [3]. Both pathways share the common intermediate coproporphyrinogen III. In contrast, archaea and sulfate-reducing bacteria employ the siroheme-dependent (SHD) biosynthesis route, which was elucidated in 2011 [4]. In the SHD pathway, uroporphyrinogen III is first converted into the name-giving intermediate siroheme in three enzymatic steps. Then, the SHD route proceeds via the intermediates 12,18-didecarboxysiroheme and iron-coproporphyrin III (FeCopro). In the last step of the SHD pathway, FeCopro is converted into heme *b* by the heme synthase AhbD. The AhbD-catalyzed reaction consists of the oxidative decarboxylation of the two propionate side chains at pyrrole rings A and B of FeCopro to the corresponding vinyl groups of heme [4]. In previous studies, it was observed that the decarboxylation of the two propionate side chains takes place sequentially with a detectable monovinyl intermediate (Figure 1) [5,6]. However, it is not known which of the propionate side chains is decarboxylated first.

AhbD belongs to the family of Radical *S*-adenosyl-l-methionine (SAM) enzymes, all of which utilize the combination of an [4Fe-4S] cluster and a cluster-bound SAM to initiate radical catalysis [7]. Three iron ions of the characteristic Radical SAM (RS) iron–sulfur cluster are coordinated by three conserved cysteine residues, which usually reside in the motif CX_3_CX_2_C. The fourth, non-cysteine bound iron ion of the cluster binds SAM via the carboxylate and amino groups of its methionine moiety [8,9]. In the first, common reaction steps of Radical SAM enzymes, the RS cluster is reduced by a physiological electron donor in vivo or by a chemical reductant in vitro. The reduced [4Fe-4S]^1+^ cluster then transfers one electron to the bound SAM, which is cleaved to methionine and a cluster-bound 5′-deoxyadenosyl radical (5′-dA^•^), an organometallic species, which is termed intermediate Ω [10]. The 5′-dA^•^ liberated from Ω then abstracts a hydrogen atom from the substrate, leading to the formation of a substrate radical and 5′-deoxyadenosine (5′-dA). After substrate radical formation, further reaction steps are different for each Radical SAM enzyme, depending on the nature of the substrate and the reaction to be catalyzed [11,12]. Within the Radical SAM family, there are several large subgroups, which share common reaction types (e.g., methyl transfer) [13], additional cofactors (e.g., cobalamin or iron–sulfur clusters) [14,15], or additional domains. An example for the latter is the subfamily of the SPASM/Twitch domain containing Radical SAM enzymes [16]. The SPASM domain was first identified as a shared motif in the Radical SAM enzymes AlbA, PqqE, anSME, and MtfC involved in the biosynthesis or maturation of subtilosin A, pyrroloquinoline quinone, anaerobic sulfatase, and mycofactocin, respectively [17]. The SPASM domain resides in the C-terminal halves of these enzymes and harbors seven to eight conserved cysteine residues that provide the ligands for two additional iron–sulfur clusters, termed auxiliary clusters. The type and function of these auxiliary clusters depend on the overall reaction of the respective enzyme. The Twitch domain is a truncated SPASM domain containing either three or four conserved cysteine residues that serve as ligands for a single auxiliary cluster [16].

AhbD belongs to the subfamily of the SPASM/Twitch domain containing Radical SAM enzymes [5,15]. In addition to the N-terminal Radical SAM domain harboring the canonical CX_3_CX_2_C motif, it exhibits a C-terminal domain with a conserved cysteine motif at the very C-terminal end, CX_2_CX_5_CX_2_CX_17_C, similar to the SPASM/Twitch motifs, and provides potential ligands for an auxiliary iron–sulfur cluster. Indeed, in previous studies of AhbDs from *Desulfovibrio vulgaris* and *Methanosarcina barkeri*, it was reported that the enzymes contain two [4Fe-4S] clusters, the RS cluster and one auxiliary cluster. The clusters were characterized using electron paramagnetic resonance (EPR) spectroscopy and their midpoint redox potentials were determined to be in the range of −340 to −400 mV [5,6]. Further, it was observed that both clusters are essential for the heme synthase activity of AhbD from *M. barkeri*. It was proposed that the auxiliary cluster might be required for the electron transfer of the remaining electron originating from the oxidative decarboxylation reaction (Figure 1) to an external electron acceptor [6].

Interestingly, in addition to the five-cysteine motif mentioned above, the AhbDs from *D. vulgaris* and *M. barkeri* contain a full SPASM motif with three more cysteine residues, and the question was raised whether a second, so far undetected, auxiliary cluster might be present in AhbD [15]. However, in previous computational structural models of AhbD, only four of the cysteine residues were located at the C-terminal end of the protein and were oriented suitably to bind a [4Fe-4S] cluster [5]. Moreover, not all of the predicted AhbD sequences contain the full SPASM motif with eight cysteine residues [own work, unpublished]. In order to clarify these contradictory findings, we decided to reanalyze the amino acid sequences of AhbDs from different organisms regarding the presence of conserved cysteine residues. Additionally, we used the AlphaFold2 structure prediction tool [18], which was not available in the past, to generate a more reliable structural model of AhbD in order to locate the position of the conserved cysteine residues within the predicted structure. We found that a subset of AhbDs indeed contains a full SPASM domain with two predicted auxiliary cluster sites. Subsequently, the number of auxiliary clusters was investigated for recombinant, purified AhbD from *M. barkeri* using several variants carrying cysteine to alanine exchanges. The variants were characterized via iron and sulfide quantification, UV/Visible absorption, and EPR spectroscopy showing that AhbD from *M. barkeri* contains two auxiliary [4Fe-4S] clusters. While the deletion of the AuxII cluster had no impact on the enzyme activity, an AhbD variant with perturbed AuxI cluster accumulated the monovinyl-intermediate. Roles in electron transfer for both auxiliary clusters as well as a role in monovinyl-intermediate release and rebinding for the AuxI cluster are discussed.

## 2. Materials and Methods

### 2.1. Chemicals

Chemicals and reagents were purchased from Carl Roth GmbH & Co. KG (Karlsruhe, Germany) or Merck KGaA (Darmstadt, Germany) unless stated otherwise. Fe-coproporphyrin III was obtained from Frontier Scientific Services Inc. (Newark, DE, USA). Enzymes, reagents, and kits for molecular biology were from New England Biolabs GmbH (Frankfurt, Germany), TaKaRa Bio Europe SAS (Saint-Germain-en-Laye, France), or Agilent Technologies (Santa Clara, CA, USA).

### 2.2. Site-Directed Mutagenesis of the ahbD Gene

For the site-directed mutagenesis of the *ahbD* gene, either a QuikChange II Site-Directed Mutagenesis Kit (Agilent Technologies) or a Q5 Site-Directed Mutagenesis Kit (New England Biolabs) was used according to the manufacturer’s instructions. The DNA template for the mutagenesis PCR was pETDuet*ahbD*/HIS [6]. For the generation of variant pETDuet*ahbD*/HIS carrying several mutated cysteine codons, which could not be incorporated into a single oligonucleotide primer, the mutagenesis was performed stepwise. The oligonucleotide primers were synthesized by Eurofins Genomics (Ebersberg, Germany) and are listed in Table S1. The introduction of mutated codons was verified with DNA sequencing.

### 2.3. Production, Purification, and Iron–Sulfur Cluster Reconstitution of Recombinant AhbD

AhbD wt and variants were produced and purified via immobilized metal chelate affinity chromatography (IMAC), as described previously [6]. Deviating from the previous purification protocol, all steps following cell harvest were conducted under anaerobic conditions in an anaerobic chamber containing 95% N_2_ and 5% H_2_ (Coy Laboratory Products, Grass Lake, MI, USA). Iron–sulfur cluster reconstitution was performed as described previously [6], but using 12 equivalents of ammonium iron citrate and lithium sulfide relative to the protein concentration.

### 2.4. Determination of Protein Concentration

The concentration of protein solutions was determined using the Bradford assay employing Quick Start Bradford 1× Dye and Quick Start Bovine Serum Albumin as the standard according to the manufacturer’s instructions (Bio-Rad Laboratories GmbH, Feldkirchen, Germany).

### 2.5. Determination of Iron and Sulfide Contents

Iron and sulfide contents of purified AhbD were determined as previously described [6].

### 2.6. Enzyme Activity and Substrate Binding Assay

Enzyme activity and substrate binding assays were performed under anaerobic conditions in an anaerobic chamber containing 95% N_2_ and 5% H_2_.

The enzyme activity assays contained 5 µM AhbD, 20 µM FeCopro, 1 mM SAM, and 1 mM sodium dithionite in reaction buffer (50 mM Tris-HCl, pH 7.5, 300 mM NaCl, 0.3% Triton-X 100). Assay mixtures were incubated at 21 °C and 100 µL samples were withdrawn after 3 and 6 h. The reaction was stopped by the addition of either 5 µL concentrated HCl (for tetrapyrrole analysis) or 5 µL formic acid (for analysis of SAM cleavage products).

Substrate binding assays were performed as described previously [6].

### 2.7. Tetrapyrrole Extraction

For tetrapyrrole extraction, 180 µL ethyl acetate was added to the 100 µL sample stopped with HCl, and the mixture was vigorously mixed for 10 min. Then, phase separation was achieved via centrifugation at 18,400× *g* for 10 min. The upper ethyl acetate fraction was taken to a new vial and the solvent was completely evaporated using an Eppendorf Concentrator Plus (Eppendorf SE, Hamburg, Germany). The extracted, dry tetrapyrroles were stored at −20 °C until HPLC analysis.

### 2.8. High-Performance Liquid Chromatography (HPLC)

For the HPLC analysis of tetrapyrroles, the extracted and dried compounds were dissolved in 100 µL 40% acetonitrile followed by the addition of 5 µL concentrated HCl. The samples were centrifuged at 15,900× *g* for 10 min and analyzed via HPLC as previously described [19]. Retention times of FeCopro and heme were determined using standard solutions of the commercially available compounds. The retention time of the monovinyl-intermediate was determined by performing an AhbD activity assay with a ferredoxin/ferrodoxin reductase system as the reducing agent instead of dithionite. In this case, the reaction was very slow, but there were only three distinct peaks in the chromatogram. Since the retention times for FeCopro and heme are known, the third peak at a retention time of 31 min was assigned to the monovinyl-intermediate, which is known to be formed during the AhbD reaction [5].

For the HPLC analysis of SAM cleavage products, the samples stopped with formic acid were centrifuged at 15,900× *g* for 10 min and analyzed using an ISAspher 100-5 C18 column (250 mm × 4 mm, ISERA GmbH, Düren, Germany) connected to a JASCO LC-4500 series system (JASCO Deutschland GmbH, Pfungstadt, Germany) with detection at 260 nm. The column was equilibrated with 98% solvent A (40 mM sodium acetate, pH 4.2) and 2% solvent B (acetonitrile). Separation of SAM and 5′-dA was achieved by applying the following method at a flow rate of 0.5 mL/min: 98% A for 2 min, gradient from 98% to 70% A within 16 min, 70% A for 2 min, gradient from 70% to 98% A within 2 min, 98% A for 18 min. Retention times of SAM and 5′-dA were determined using standard solutions of commercially available SAM (Sigma-Aldrich, Taufkirchen, Germany) and 5′-dA (Sigma-Aldrich).

### 2.9. EPR Spectroscopy

EPR measurements were conducted with an EMX 6/1 ESR spectrometer (Bruker, Karlsruhe, Germany) operating at X-band in combination with a Bruker ER4012 cavity. The sample temperature was controlled with an ESR-900 helium flow cryostat (Oxford Instruments, Abingdon, UK). The magnetic field was calibrated using a Bruker strong pitch standard. EPR conditions were: microwave frequency, 9.44 GHz; modulation amplitude, 0.60 mT; modulation frequency, 100 kHz; time constant, 0.164 s; scan rate, 17.9 mT/min. The samples were measured at 13 K and 40 K at different microwave powers. These values are provided in the figure caption. The EPR spectra were simulated by using the Matlab toolbox Easyspin [20]. The simulations were performed by using a Gaussian g-strain model.

## 3. Results

### 3.1. A Subset of AhbD Sequences Displays a Full SPASM Motif with Eight Conserved Cysteines

**Figure 2:**
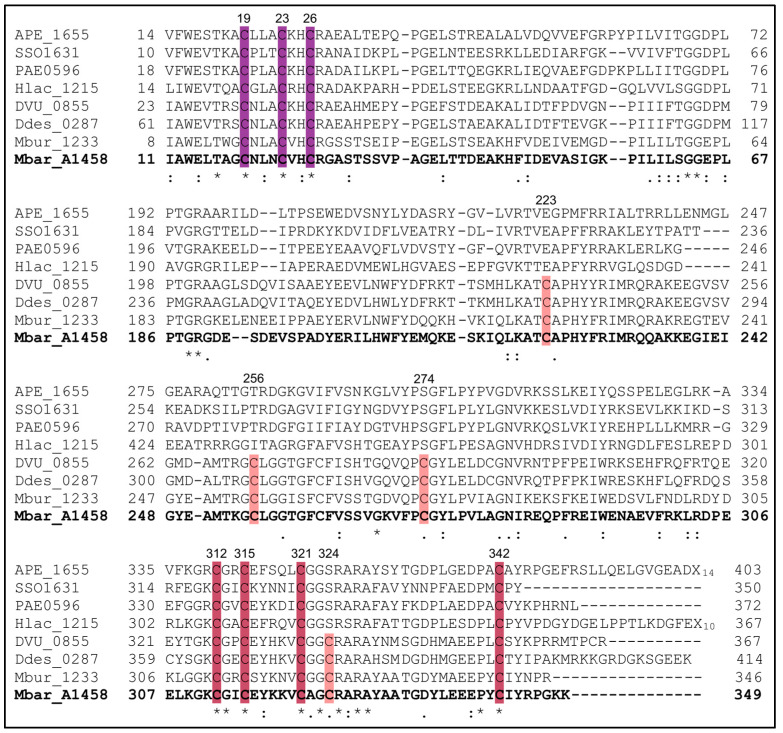
Amino acid sequence alignment of AhbD sequences from different archaea and sulfate-reducing bacteria. Note the gaps between the first and second row, as well as the second and third row. The complete alignment, including additional sequences, is shown in Figure S1. Conserved N-terminal cysteine residues coordinating the RS cluster are highlighted in violet purple, C-terminal cysteine residues in raspberry, and partially conserved cysteine residues of the full SPASM motif in salmon. Sequences are specified by gene numbers. Organism abbreviations are as follows: APE, *Aeropyrum pernix*; SSO, *Sulfolobus solfataricus*; PAE, *Pyrobaculum aerophilum*; Hlac, *Halorubrum lacusprofundi*; DVU, *Desulfovibrio vulgaris*; Ddes, *Desulfovibrio desulfuricans*; Mbur, *Methanococcoides burtonii*; Mbar, *Methanosarcina barkeri*. Asterisks highlight strictly conserved amino acid residues. The sequence of AhbD from *M. barkeri* is in bold.

In order to clarify the number of conserved cysteine residues in AhbD, a total of 39 AhbD sequences from different organisms were chosen and compared using the Clustal Omega tool [21]. In total, 28 AhbD sequences were retrieved from archaeal species based on their identification by Storbeck et al. [22] and comprised 20 sequences from non-methanogenic and 8 from methanogenic archaea. The remaining 11 sequences represented AhbD proteins from different sulfate-reducing bacteria. The inspection of the amino acid sequence alignment, of which a shortened version is shown in Figure 2 (see Figure S1 for the full version), revealed that the AhbD sequences can be divided into two major groups with respect to their cysteine content. While all AhbD sequences included in the study exhibited the conserved Radical SAM motif CX_3_CX_2_C at the N-terminus and the conserved CX_2_CX_5_CX_20-38_C motif at the C-terminus, both previously recognized [5,6], only a subset of AhbD sequences displayed a full SPASM motif containing overall eight conserved cysteine residues within the C-terminal half of the protein. The full SPASM motif CX_20-33_CX_17_CX_36-37_CX_2_CX_5_CX_2_CX_17-35_C was present in the AhbD proteins from methanogenic archaea and sulfate-reducing bacteria. AhbD sequences from non-methanogenic archaea did not display the full SPASM motif, except for the sequence of AhbD from *Metallosphaera sedula*.

In the AhbDs from non-methanogens, four cysteine residues of the full SPASM motif were not present. Instead, three of these positions (C223, C274, C324, *M. barkeri* numbering) were occupied by conserved glutamate and serine residues. In contrast, the amino acid position corresponding to C256 did not represent a conserved residue in non-methanogenic archaeal AhbDs.

### 3.2. The AlphaFold2 Model of AhbD from M. barkeri Suggests the Presence of Two Auxiliary Iron–Sulfur Clusters

**Figure 3:**
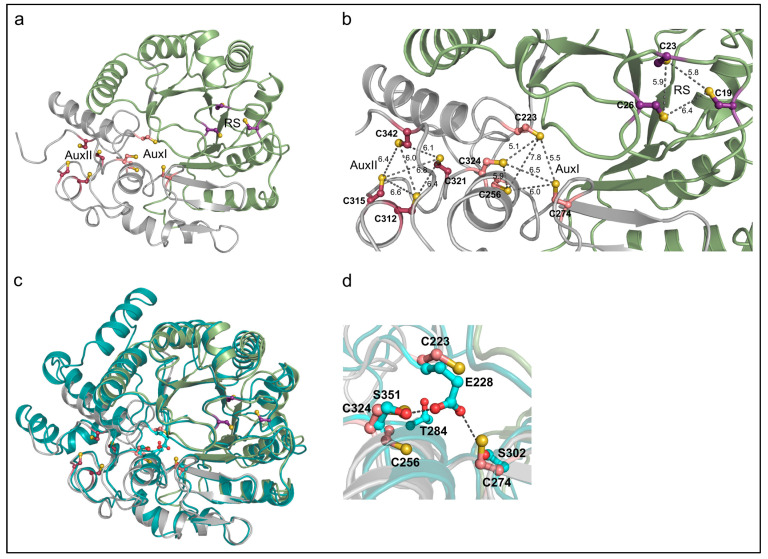
Computational structural models of AhbD. (**a**), Model of AhbD from *M. barkeri*. The three-quarter triose phosphate isomerase (TIM) barrel, representing the characteristic Radical SAM domain, is shown in green and the C-terminal part of AhbD, including the predicted SPASM domain, is shown in gray. Conserved N-terminal cysteine residues coordinating the RS cluster are shown in stick-and-ball representation in violet purple, C-terminal cysteine residues in raspberry, and partially conserved cysteine residues of the full SPASM motif in salmon. (**b**), Same as in (**a**), with focus on the conserved cysteine residues. Dashed lines indicate sulfur to sulfur distances as labeled. The distance measurement was performed with PyMOL. (**c**), Superposition of the structural model of AhbD from *M. barkeri* (colors as in (**a**)) with that of AhbD from *A. pernix* (teal). Two serine, one glutamate, and one threonine residue of AhbD from *A. pernix* are shown in stick-and-ball representation in cyan. (**d**), Same as in (**c**), with focus on the cysteine residues conserved in AhbDs from methanogens and sulfate-reducing bacteria, but not present in AhbDs from non-methanogenic archaea. In the latter, two conserved serine and one conserved glutamate replace three of the cysteine residues occupying similar positions. Dashed lines indicate potential hydrogen bonds. The figure was prepared using PyMOL (Schrödinger LLC., New York, NY, USA; version 2.3.0).

As described in the Introduction, previous computational models of the AhbD structure only placed the four cysteine residues of the C-terminal CX_2_CX_5_CX_20-38_C motif (Figure 2, raspberry) in a suitable orientation for the ligation of an auxiliary iron–sulfur cluster. In contrast, for the other four conserved cysteine residues of the SPASM domain (Figure 2, salmon), such an orientation was not observed. With the advent of AlphaFold, more reliable protein structure prediction is possible [23]. Therefore, we created an AlphaFold2 model of AhbD from *M. barkeri* using the AlphaFold2.ipynb notebook [18] and analyzed the structural model for the location and orientation of the conserved cysteine residues (Figure 3a).

As observed in previous AhbD models, the cysteine residues of both the Radical SAM motif and the C-terminal motif were located and oriented in a manner suitable for iron–sulfur cluster ligation. Moreover, in the new AlphaFold2 model, the four additional cysteine residues within the SPASM domain were also properly positioned for the accommodation of an auxiliary cluster. The distances between the sulfur atoms in each cluster binding site mostly ranged from about 6.0 to 6.8 Å (Figure 3b), in agreement with the distances found in typical [4Fe-4S] cluster proteins. Only three outliers with distances of 5.1, 5.5, and 7.8 Å were observed. Based on the amino acid sequence alignment described above and the AlphaFold2 model, we hypothesized that AhbDs from methanogenic archaea and sulfate-reducing bacteria indeed carry two auxiliary iron–sulfur clusters, as observed in other SPASM-domain-containing Radical SAM enzymes such as anSME, PqqE, MftC, and SuiB [24,25,26,27,28]. An amino acid sequence comparison of these well-characterized enzymes with AhbD supports this hypothesis, since seven out of the eight cysteine residues of the AhbD SPASM motif aligned almost perfectly with homologous residues of anSME, PqqE, MftC, and SuiB (Figure S2). The only exception was C223 of AhbD from *M. barkeri*. However, the position of this cysteine within the AhbD sequence is similar to that of one cysteine ligand of the AuxI cluster in SuiB. Indeed, the search for structural relatives of the AhbD model using the DALI server [29] revealed the SuiB structure as the first hit. Therefore, for AhbD from *M. barkeri*, cysteine residues C223, C256, C274, and C324 (Figure 3a,b, salmon) are proposed to coordinate the auxiliary cluster AuxI, and cysteine residues C312, C315, C321, and C342 (Figure 3a,b, raspberry) the auxiliary cluster AuxII.

As described above, AhbDs from non-methanogenic archaea do not display the full SPASM motif. The superposition of the AhbD model from *M. barkeri* with the AlphaFold2 model of AhbD from *Aeropyrum pernix* (Uniprot entry Q9YBE2) shows that both structural models are highly similar except for two additional, peripheral helices in the AhbD from *A. pernix* (Figure 3c). In the model of the latter, three of the cysteine residues that potentially coordinate the proposed AuxI cluster in AhbD from *M. barkeri* are replaced by one glutamate and two serine residues (Figure 3d), as already observed in the amino acid sequence alignment. The carboxylate oxygen atoms of the glutamate might form hydrogen bonds with the hydroxyl groups of the serine residues (O-O distances of 2.5 and 3.7 Å).

### 3.3. AhbD Variants Support the Presence of Two Auxiliary [4Fe-4S] Clusters

#### 3.3.1. Production and Purification of AhbD Cluster Variants Carrying a Single Cluster

**Figure 4:**
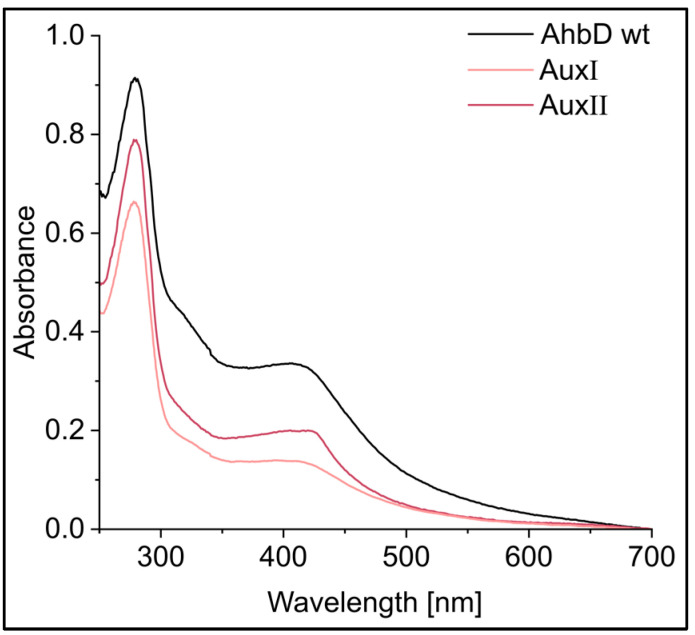
UV/Visible absorption spectra of purified, reconstituted AhbD wt and the AhbD variants AuxI and AuxII.

In order to test the hypothesis that AhbD from *M. barkeri* is a true SPASM domain containing a Radical SAM enzyme carrying two auxiliary iron–sulfur clusters, we generated two AhbD variants. In both variants, the cysteine residues C23 and C26 were replaced by alanine residues, resulting in the deletion of the RS cluster. Additionally, in one of the variants, C312 and C315, coordinating the AuxII cluster, were also exchanged with alanine residues. The resulting variant was termed AhbD AuxI, since it should carry the potential AuxI cluster as the only remaining cluster. The second variant carried cysteine to alanine exchanges for C256, C274, and C324 in addition to the exchanges of C23 and C26. Therefore, this variant should carry the AuxII cluster as the only remaining cluster and was accordingly termed AhbD AuxII. Both variants, as well as wild type (wt) AhbD, were heterologously produced in *E. coli* BL21(DE3) and purified by IMAC. After purification, the iron–sulfur clusters were chemically reconstituted. While AhbD wt exhibited a dark brown color, the protein solutions of both variants were light brown. The iron–sulfur cluster contents of the preparations were analyzed with UV/Visible absorption spectroscopy and determination of iron and sulfide contents. As shown in Figure 4, the UV/Visible spectra of all three proteins displayed a broad absorption band around 410 nm, typical for proteins carrying [4Fe-4S] clusters.

The ratio A_410_/A_280_ was determined to be 0.37 for AhbD wt, 0.21 for variant AuxI, and 0.25 for variant AuxII, indicating a lower iron–sulfur cluster content for the variants compared with the wt enzyme. Table 1 shows the iron and sulfide content of the preparations. While the iron and sulfide content of AhbD wt supports the presence of up to three iron–sulfur clusters, the content determined for both variants indicates the loss of two clusters. Importantly, both variants still contained iron and sulfide, in agreement with the presence of one iron–sulfur cluster.

#### 3.3.2. EPR Spectroscopy Demonstrates the Presence of Two Auxiliary [4Fe-4S] Clusters

**Figure 5:**
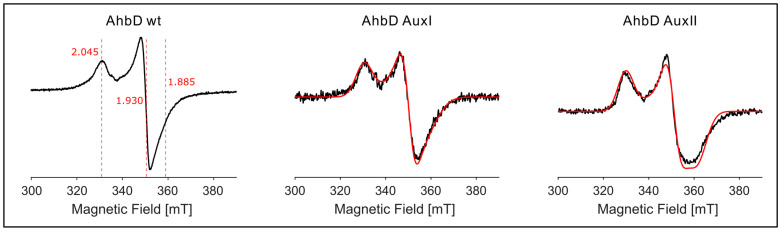
EPR Cw X-band measurement (black) and the corresponding simulations (red) for the reconstituted, dithionite-reduced AhbD proteins. The EPR spectra were recorded at *T* = 13 K and *P*_MW_ = 1 mW. All spectra were normalized to their maximum for comparison. The other experimental EPR parameters and the simulation parameters are given in the main text. (**Left**): AhbD wt; (**middle**): AhbD variant AuxI; (**right**): AhbD variant AuxII.

In order to determine the type of the auxiliary clusters, i.e., [4Fe-4S] or [2Fe-2S], the preparations were analyzed using EPR spectroscopy. The spectrum of purified and reconstituted AhbD wt (dithionite-reduced) showed a very weak and broad, slightly rhombic signal at 40 K and 5 mW microwave power. The signal was saturated already at 20 mW power. An identical spectrum of much higher intensity was obtained at 13 K and 1 mW microwave power, indicating the presence of a [4Fe-4S] cluster with principal g-values of $\left[g_{x},\;g_{y},\;g_{z}\right]=[2.045,\;1.93,\;1.885]$ (Figure 5). A similar spectrum with similar principal g-values $\left[g_{x},\;g_{y},\;g_{z}\right]=\left[2.05,\;1.93,\;1.89\right]$ was obtained at 13 K and 1 mW power with the reconstituted preparation of the AhbD AuxI variant. However, this signal was not detectable at 40 K and 5 mW. Similarly, for the reconstituted preparation of the AuxII variant, the spectrum recorded at 40 K and 5 mW revealed no signals, whereas measurements at 13 K and 1 mW power resulted in a similar spectrum of a tetranuclear cluster with a more rhombic contribution. The spectrum was simulated with $\left[g_{x},\;g_{y},\;g_{z}\right]=[2.05,\;1.93,\;1.87]$. Importantly, EPR spectra recorded for the AuxI and AuxII preparations before iron–sulfur cluster reconstitution representing the physiological forms exhibited identical g-values to their reconstituted counterparts (Figure S3). EPR spectra measured for the preparations without dithionite showed only very minor amounts of [3Fe-4S] clusters (Figure S4). In the g~4 region of the spectra, small signals for “free” Fe^3+^ were detected, typical for preparations of iron–sulfur proteins (Figure S4). Thus, the EPR experiments show that both variants, AuxI and AuxII, contain a single tetranuclear iron–sulfur cluster with highly similar symmetry. In conclusion, these data suggest that AhbD from *M. barkeri* contains three [4Fe-4S] clusters, the RS clusters as well as two auxiliary clusters.

### 3.4. Role of the Auxiliary Clusters for AhbD Activity

#### 3.4.1. Production and Purification of AhbD Cluster Variants Lacking AuxI or AuxII

In order to further investigate the individual roles of the two auxiliary clusters of AhbD, we generated variants in which either one of the two Aux clusters was deleted or at least perturbed. In the case of AhbD variant RS/AuxI, cysteine residues C312, C315, and C321 were exchanged with alanine residues. Thus, this variant was expected to contain the RS and AuxI cluster and to be devoid of the AuxII cluster. In order to obtain a variant RS/AuxII lacking the AuxI cluster, we initially replaced three (C256, C274, and C324) or two (C256 and C324) cysteines with alanine residues. Unfortunately, these variants showed an elevated tendency for aggregation and were almost completely inactive in terms of both SAM cleavage and heme synthase activity, suggesting that the amino acid exchanges disturbed the structural integrity of the (active site of the) protein. Therefore, only C324 was exchanged with an alanine residue. Although this single-cysteine residue exchange might not result in complete deletion of the AuxI cluster, one would expect at least a perturbation of the cluster, such as the loss of one iron ion or a change in redox potential. The cluster variants were produced and purified followed by iron–sulfur cluster reconstitution. The UV/Visible absorption spectra displayed broad bands around 410 nm and the ratio A_410_/A_280_ was determined to 0.36 and 0.3 for variants RS/AuxI and RS/AuxII, respectively. The determined iron and sulfide content of the preparations are listed in Table 1. Altogether, these results suggest that both variants carried two clusters, while one cluster was lacking, as intended.

#### 3.4.2. The Auxiliary Clusters Are Not Required for FeCopro Binding

**Figure 6:**
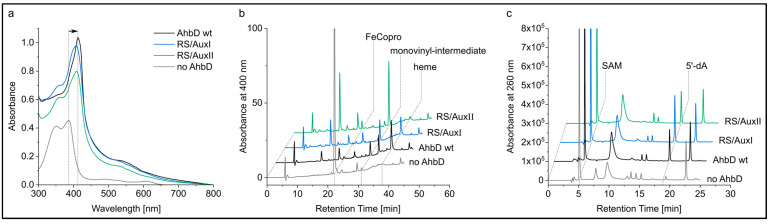
Substrate binding and enzyme activity assays. (**a**), UV/Visible absorption spectra of FeCopro binding assays recorded after 24 h of incubation. The arrow indicates the shift of the Soret band of unbound FeCopro at 387 nm (gray) to 413 nm upon binding to AhbD wt. (**b**), HPLC separation of tetrapyrroles extracted from enzyme activity assay mixtures after 3 h of incubation. FeCopro eluted at a retention time of 22.1 min, the monovinyl-intermediate at 31 min, and heme at 37.8 min, as determined with standard solutions (see Section 2). Peaks at other retention times were not further investigated. (**c**), HPLC separation of SAM cleavage products formed after 3 h of incubation. SAM and 5′-dA eluted at retention times of about 5 and 18.9 min, respectively, as determined with standard solutions. Peaks at other retention times were not further investigated.

In order to test a potential involvement of the auxiliary clusters in substrate binding, a previously established FeCopro binding assay was performed with AhbD wt and the variants RS/AuxI and RS/AuxII. In this assay, AhbD is mixed with equimolar amounts of the substrate FeCopro and UV/Visible absorption spectra are recorded after 24 h of incubation at room temperature. FeCopro binding to AhbD is accompanied by a shift of the Soret band to longer wavelengths [6]. As shown in Figure 6a, the Soret band of unbound FeCopro at 387 nm shifted to 413 nm in the presence of AhbD wt. Similar shifts were observed for both AhbD variants. Here, the Soret band was observed at 408 nm with shoulders around 390 and 360 nm, the latter suggesting the presence of remaining unbound FeCopro. Overall, the substrate binding assay indicates that both AhbD variants were able to bind FeCopro, possibly with lower affinity than the wt enzyme, and that neither the AuxI nor the AuxII cluster is required for substrate binding.

#### 3.4.3. AhbD Variant RS/AuxII Accumulates the Monovinyl Intermediate

Enzyme activity assays were performed with AhbD wt and the two variants RS/AuxI and RS/AuxII. In these assays, AhbD (5 µM) was incubated with the substrate FeCopro (20 µM), SAM (1 mM), and sodium dithionite (1 mM) as the reducing agent. After incubation for 3 and 6 h at room temperature, samples were withdrawn from the assay mixture and the reaction was stopped as described in Section 2. After solvent extraction, the tetrapyrroles within the samples were analyzed using HPLC. Similarly, the formation of 5′-dA was also analyzed using HPLC. After 3 h of incubation, the substrate FeCopro was almost completely consumed by AhbD wt and the variant RS/AuxI. The formation of the reaction product heme as well as the monovinyl-intermediate was observed in both cases (Figure 6b). After 6 h, the heme/monovinyl-intermediate ratio had increased for both enzymes (Figure S5). In contrast, for the AhbD variant RS/AuxII, almost no heme formation was observed after 3 and 6 h, although the monovinyl-intermediate was formed and FeCopro was completely consumed after 6 h. The HPLC analysis of the assay mixtures for 5′-dA revealed the formation of this reaction byproduct for all three enzymes (Figure 6c and Figure S5).

## 4. Discussion

In this study, we demonstrate that the heme synthase AhbD from *M. barkeri* contains two auxiliary [4Fe-4S] clusters and, therefore, is a true member of the Radical SAM subfamily exhibiting a SPASM domain. In previous biochemical characterizations of AhbD proteins, including our own, one of the auxiliary iron–sulfur clusters was overlooked and AhbD was described as a Radical SAM enzyme containing only one auxiliary cluster [5,6]. At the time, this finding was supported by the fact that not all of the eight cysteine residues of the SPASM domain are strictly conserved in AhbD sequences from different archaea. Moreover, previous structural models of AhbD also indicated the presence of only one auxiliary cluster. Here, we reanalyzed the iron–sulfur cluster content of AhbD from *M. barkeri* using improved structure-prediction tools as well as biochemical and spectroscopic methods. Thereby, we demonstrate that the protein indeed contains two auxiliary [4Fe-4S] clusters, AuxI and AuxII, in addition to the RS cluster. In previous preparations, AhbD was purified under aerobic conditions followed by anaerobic iron–sulfur cluster reconstitution [6]. In contrast, in this study, the purification of AhbD was conducted completely under anaerobic conditions, leading to improved iron–sulfur cluster content.

Within the subfamily of SPASM-domain-containing Radical SAM enzymes, variations in the cluster type and in the cluster coordination of the auxiliary clusters exist. Most members of the family, such as the well-characterized anSME, CteB, MftC, and SuiB, contain two auxiliary [4Fe-4S] clusters [27,28,30,31]. While the AuxII cluster in these examples is always coordinated by four cysteine residues, crystal structures revealed that the coordination of the AuxI cluster is achieved by either four (anSME, SuiB) [28,30] or three cysteine residues (CteB) [31]. In the latter case, the open coordination site at the non-cysteine-coordinated iron ion can participate in substrate binding, as demonstrated for a subgroup of SPASM-domain-containing peptide maturases introducing C–S bonds during the biosynthesis of ranthipeptides [32]. Since the enzymes mentioned above all catalyze oxidative reactions, a proposed common role for the auxiliary clusters is electron transfer from the substrate radical to either an external electron acceptor or back to the RS cluster [27,33,34]. In addition to the SPASM-domain-containing enzymes containing two auxiliary [4Fe-4S] clusters with cysteine ligands, PqqE represents an exception in that its AuxII cluster is coordinated by three cysteine residues and an aspartate residue [26]. Moreover, the AuxI cluster of PqqE is proposed to be a [2Fe-2S] cluster with four cysteine ligands potentially involved in electron shuttling from the substrate radical to the RS cluster [35]. In terms of cluster type and cluster coordination, AhbD from *M. barkeri* belongs to the subgroup of SPASM-domain-containing enzymes carrying two [4Fe-4S] clusters with full cysteine coordination and, therefore, resembles anSME and SuiB. The presence of four cysteine ligands for the AuxI cluster in AhbD is proposed based on the amino acid sequence alignment shown in Figure S2 and the high similarity in cysteine positions compared with those in SuiB. The assumption of an AuxI cluster coordinated by four cysteine residues in AhbD excludes a role of this cluster in direct substrate binding. This suggestion is supported by our substrate binding assay showing that FeCopro is bound by the AhbD RS/AuxII variant. Moreover, the first decarboxylation reaction is carried out by variant RS/AuxII (Figure 6b), demonstrating that binding of the initial substrate FeCopro is not impaired. However, the AuxI cluster appears to play a role in the second decarboxylation reaction converting the monovinyl-intermediate into the final product heme, an activity that is almost completely abolished in the RS/AuxII variant. Although the precise role of the AuxI cluster cannot be deduced from our experiments, two potential functions remain. Either the AuxI cluster might serve as an electron acceptor for the remaining electron of the oxidative decarboxylation reaction, or it might be involved in the release and rebinding as well as correct orientation of the monovinyl-intermediate for the second decarboxylation to take place. Although both of these roles appear attractive, it has to be considered in this context that not all AhbDs from different organisms contain the AuxI cluster. Therefore, whatever role the AuxI cluster in AhbD from *M. barkeri* adopts, it has to be taken by the conserved amino acid residues present in AhbDs lacking the AuxI-coordinating cysteine residues. The role of the AuxII cluster in AhbD remains enigmatic. Under the assay conditions employed in this study, the RS/AuxI variant exhibited heme synthase activity similar to that of AhbD wt. This is in contrast to other SPASM-domain-containing Radical SAM enzymes such as, e.g., MftC and PqqE, in which the AuxII cluster was observed to be required for the formation of the final products, although SAM cleavage was not impaired [27,35]. Altogether, the elucidation of the roles of the auxiliary clusters in AhbD has to be addressed in future studies.

Although we demonstrated the presence of two auxiliary [4Fe-4S] clusters in AhbD from *M. barkeri*, the amino acid sequence analysis of AhbDs from different organisms indicates that only the AuxII cluster is conserved. Most of the AhbD sequences from non-methanogenic archaea do not contain the cysteine residues coordinating the AuxI cluster. Instead, the respective positions are occupied by two serine and one glutamate residue, which appear to be conserved in AhbDs from non-methanogenic archaea. Thus far, none of the AhbDs containing only AuxII have been studied biochemically. Therefore, the predicted heme synthase activity must be tested for these enzymes. The comparison of the enzymatic properties of the two AhbD subgroups might provide further insights into the role of the AuxI cluster.

## Figures and Tables

**Table 1:** Iron and sulfide contents of AhbD wt and variants.

AhbD Variant	Cys to Ala	Iron (mol/mol AhbD) ^1^	Sulfide (mol/mol AhbD) ^1^
wt		11.8 ± 1.5	10.1 ± 1.1
AuxI	23 + 26 + 312 + 315	2.7 ± 1.4	3.2 ± 0.7
AuxII	23 + 26 + 256 + 274 + 324	2.4 ± 0.8	3.4 ^2^
RS/AuxI	312 + 315 + 321	8.3 ± 1.0	5.6 ± 1.0
RS/AuxII	324	7.2 ± 2.7	4.5 ± 1.8

^1^ Mean values with standard deviation obtained from at least two independent enzyme preparations are given. ^2^ Value of only one preparation.

## Data Availability

The data presented in this study are available on request from the corresponding author.

## Supplementary Materials

The following supporting information can be downloaded at: https://www.mdpi.com/article/10.3390/biom13081268/s1, Figure S1: Amino acid sequence alignment of AhbD sequences from different archaea and sulfate-reducing bacteria; Figure S2: Amino acid sequence alignment of AhbD with the SPASM-domain-containing Radical SAM enzymes anSME, SuiB, PqqE, and MftC; Figure S3: EPR Cw X-band measurements for the dithionite-reduced AhbD variants AuxI and AuxII recorded before iron–sulfur cluster reconstitution and after reconstitution; Figure S4: EPR Cw X-band measurements for the oxidized AhbD variants AuxI and AuxII, recorded before and after iron–sulfur cluster reconstitution; Figure S5: HPLC analysis of enzyme activity assay mixtures containing AhbD wt and variant RS/AuxI or RS/AuxII; Table S1: Oligonucleotide primers for site-directed mutagenesis of the *ahbD* gene.
