# Supplementary material for: The Radical SAM Heme Synthase AhbD from Methanosarcina barkeri Contains Two Auxiliary [4Fe-4S] Clusters
Source: Biomolecules. 2023 Aug 18;13(8):1268. doi: 10.3390/biom13081268 (PMC10452713; doi:10.3390/biom13081268)
Supplement: Supplementary file 1 [file biomolecules-13-01268-s001.zip › biomolecules-2527331-supplementary.pdf]

Figure S1

```
Igni_0397      -----------------------------------------------------------M   1
APE_1655       -----------------------------------------------MRGRWPYEEKPLI  13
Hbut_0035      ------------------------------------MKVGSEHSGYPLHRTWPFERNPLL  24
ST0127         ---------------------------------------------------MPFEDAPHL   9
M1425_1048     ---------------------------------------------------MPFENAPHL   9
SSO1631        ---------------------------------------------------MPFENAPHL   9
SSO1840        ---------------------------------------------------MPFENAPHL   9
Cmaq_1900      -------------------------------------------------MPLDYSQRPLL  11
Pisl_0113      -------------------------------------------MREVKKLIERFHSAPLI  17
Tneu_1901      -------------------------------------------MRGIRELIERFHSAPLI  17
Pcal_1716      -------------------------------------------MRSVEELIRRFHEAPLL  17
PAE0596        -------------------------------------------MRNVQELIRRFHEAPLL  17
Pars_2255      -------------------------------------------MRNVQELIRRFHEAPLL  17
Msed_0512      ------------------------------------------------------MVAPYV   6
AF_2413        ------------------------------------------------------MDTPFI   6
NP_1546A       ---------------------------------------MSTGAPIPSPRDIDTSQRPFV  21
VNG_1185G      ------------------------------------------------MTPVDTSERPVV  12
Hlac_1215      -----------------------------------------------MFDNLDTDRRPLV  13
rrnAC3489      -----------------------------------------------MRGKLDLGEQPLV  13
Hmuk_1679      ------------------------------------------------MRGVDVEERPLV  12
LRZ99_07755    ----------------------------------------------------------MI   2
Memar_0879     ---------------------------------------------MRNSIGDSGKSEPTL  15
Mboo_0958      --------------------------------------------------MNDTSGAPRI  10
Mpal_2626      ---------------------MI------ENLTKADKELGECGIRTDVVKGIPGGAPPRI  33
Mthe_1135      ---------------------------------------------------------MLL   3
C0624_00890    ------------------------------------------------MSTSQEEFIPKW  12
Dace_3005      ------------------------------------------------MADQEEKFIPKW  12
CSA32_02305    -----------------------------------------------------MNFIPKW   7
DSY57_04415    -----------------------------------------------------MDFEPKW   7
B5M56_04105    ----------------------------MSQ-----KHPA--------GEHNGKKAELRL  19
DSCW_66730     ----------------------MNPHPPMYQGSPPHG------------RGSGKNDTLRL  26
DEB50_09370    ---------------------MAHPH-----KTHPPGHGG--------PHAAGKNNTLRL  26
CSA25_02885    ---------------------MAHPH-----KTPPHGHAA--------PHAGGKKDILRL  26
DVU_0855       --------------------------------------MGAHPTAHGPRTLEDGSPTCKL  22
Ddes_0287      MSKHMHHAGGHPGGMPGSAGNPISGHPGQAEDGKHRGHPGGHGMSAPLRTLEDGSPACRL  60
Mbur_1233      -----------------------------------------------------MVKPPRL   7
Mbar_A1458     --------------------------------------------------MIAMTNAPRL  10
MA_0573        --------------------------------------------------MIAMTNPPRL  10
MM_1737        --------------------------------------------------MIAMTNPPRL  10
```

continued on next page

```
Igni_0397      VFWETTKACKLVCKHCRAEAIEEPL-PDELTHEESLALIDQITEFGRPYPHLIMTGGDVL   60
APE_1655       VFWESTKACLLACKHCRAEALTEPQ-PGELSTREALALVDQVVEFGRPYPILVITGGDPL   72
Hbut_0035      VFWETTKACMLACKHCRASAILKSL-PGELTTEEAYKLIDDVAAFGQPYPILVLTGGDPL   83
ST0127         VFWEVTKACPLTCKHCRANAIDKPL-PGELTTEEGKKLLEEISQFGK--VVVVFTGGDPL   66
M1425_1048     VFWEVTKACPLTCKHCRANAIDKPL-PSELNTEESRKLLEDIARFGK--VVIVFTGGDPL   66
SSO1631        VFWEVTKACPLTCKHCRANAIDKPL-PGELNTEESRKLLEDIARFGK--VVIVFTGGDPL   66
SSO1840        VFWEVTKACPLSCKHCRANAIDKPL-PGELSTEESKKLLEDIARFGK--VVIVFTGGDPL   66
Cmaq_1900      VFWETTKACPLSCRHCRANAILKPL-PGELSTDEGKRLIEQLPEFGKPTPVLILTGGDPL   70
Pisl_0113      VFWESTKACPLACKHCRADAILKPL-PGELNTEEGKRLIEQVASFGDPKPLLVITGGDPL   76
Tneu_1901      VFWESTKACPLACKHCRADAILRPL-PGELNTEEGKRLIEQVASFGDPKPLLVITGGDPL   76
Pcal_1716      VFWESTKACPLACKHCRADAILKPL-PGELSTEEGKRLIEQVAEFGDPKPLLIITGGDPL   76
PAE0596        VFWESTKACPLACKHCRADAILKPL-PGELTTQEGKRLIEQVAEFGDPKPLLIITGGDPL   76
Pars_2255      VFWESTKACPLACKHCRADAILKPL-PGELTTQEGKRLIEQVAQFGDPKPLLIITGGDPL   76
Msed_0512      VVLESTKACDLACKHCRAKAIPNRL-PGELTTEEVKSLVDDLASSGV--KLFVISGGDAL   63
AF_2413        VFWELTRACMLACKHCRAKAIRKRH-PDELTTEECFNVIDQLSEFNP-KPLLIITGGDPL   64
NP_1546A       LIWEVTQACELACDHCRADAKPSRH-PDELTTAEGKRLLDSAAEFGD-GQLVVLSGGDPM   79
VNG_1185G      LVWEVTQACALACDHCRASARPQRH-PSELSTAEGKQLLTDAAAFGD-GQLVVLSGGDPL   70
Hlac_1215      LIWEVTQACGLACRHCRADAKPARH-PDELSTEEGKRLLNDAATFGD-GQLVVLSGGDPL   71
rrnAC3489      LIWEVTQACELACKHCRADAQPRRH-PDELSTAEGKALLDQASEFGD-GQLVVLSGGDPL   71
Hmuk_1679      LIWELTQACGLTCKHCRADARPERH-PDELTTAEGKRLLDQAADFGD-GQLVVLSGGDPL   70
LRZ99_07755    VSWNTTNACNMYCQHCYRDAGAKA--DEELNTVEGKALITEIAKAGF--KIMIFSGGEPL   58
Memar_0879     ISWNVTYRCNLRCAHCYMDAGDGGG-ALELSTSEAKMLIDQVKQAGS--PVLILSGGEPL   72
Mboo_0958      ISWNLTLRCPLKCSHCYVDAGTREA-PGVLSTDEALGVLDRIRATGT--PVVVLSGGEPL   67
Mpal_2626      ISWNITLRCPLKCAHCYVDAGEKEA-DRVLSTQEALSVIDQIRAIGK--PVVVLSGGEPL   90
Mthe_1135      VAWESTGACNLSCSYCRASAGPAPS-GDELSTGEIKGLIKEIAPMGA---MLIISGGEPL   59
C0624_00890    IAWETTQRCNLNCVHCRCSSDMDAA-EGDFNTEEAFKLIDDICEVSK--PVMVLSGGEPL   69
Dace_3005      IAWETTRRCNLTCVHCRCSSDMEAA-SGDFTTEEAFKMIDDICEVSK--PVMVLSGGEPL   69
CSA32_02305    IAWEITRRCNLQCVHCRSSSQIEVVEHPDFSYEEAKRVLRDIHSYAN--PVIVLSGGEPL   65
DSY57_04415    IAWETTRRCNLKCVHCRSSSKLEIKGHPDFSLDEAKRVLDDIHSYAD--PVIVLSGGEPL   65
B5M56_04105    VAWETTRNCNLSCRHCRASATMGPY-SGELDTDASFRLLDQIAETGS--PIVILTGGEPL   76
DSCW_66730     VAWETTRNCNLSCVHCRAAATKGPY-DGELDTDQSFALLDQIAEVGR--PIIILTGGEPL   83
DEB50_09370    VAWETTRRCNLTCKHCRAAAEDHVY-NDELTTEESFRLLDQIREVGQ--PIIILTGGEPL   83
CSA25_02885    VAWETTRRCNLTCKHCRAAAQDHVY-KDELTTQESFTLLDQIREVGQ--PIIILTGGEPL   83
DVU_0855       IAWEVTRSCNLACKHCRAEAHMEPY-PGEFSTDEAKALIDTFPDVGN--PIIIFTGGDPM   79
Ddes_0287      IAWEVTRSCNLACKHCRAEAHPEPY-PGELSTAEAKALIDTFTEVGK--PIIIFTGGDPM  117
Mbur_1233      IAWELTWGCNLACVHCRGSSTSEIP-EGELSTSEAKHFVDEIVEMGD--PILILTGGEPL   64
Mbar_A1458     IAWELTAGCNLNCVHCRGASTSSVP-AGELTTDEAKHFIDEVASIGK--PILILSGGEPL   67
MA_0573        IAWELTAGCNLNCVHCRGASTSSVP-EGELTTEEAKHFIDEVVELGK--PILILSGGEPL   67
MM_1737        IAWELTAGCNLNCVHCRGASTSSVP-EGELTTEEAKHFIDDVVELGK--PILILSGGEPL   67
               :  : *  * : * :*   :         :       .:            .:::**: :
```

continued on next page

```
Igni_0397      MRKDFWDLARYSISKGIRTLVAPSVTPLLTEEKIKKMKEIGIIGMSLSLDGARAETHDSI  120
APE_1655       MRRDFWRILEYAVSQGLRVAVAPSVTPLLTREVVRRMARMGVARISISIDSGLPEVHDAI  132
Hbut_0035      LRSDIWDIIAYAKGKGLRLAVAPAVSPNLTEDKVKKLAELGVDGVSISLDGSRPEIHDGI  143
ST0127         SRDDIFELMDYAKSLGLIVSIAPAPSYRLNEDTIKNIKN-SALYMSISLDGYKPETHDWL  125
M1425_1048     SRSDIFELMEYAKSLGLVVSIAPSPSHRLDDETMKMISN-YARYMSISLDGATPQTHDWL  125
SSO1631        SRSDIFELMEYAKSLGLVVSIAPSPSHRLDDETMKIISN-YARYMSISLDGATSQTHDWL  125
SSO1840        SRSDIFEIMEYAKSLGLIVSIAPSPSYRLRDETMKMISN-YARYMSISIDGATSQTHDWL  125
Cmaq_1900      MREDIFELIDYAKSLNVPVAVSPTVSEKLLSDNVID-ELRRVSSVSVSLDGASPTTHEYI  129
Pisl_0113      MRNDLFDLIDYANQLGVPTSLAPAVSPNLNQEALKAVREHGVKSISISLDGAREETHDEL  136
Tneu_1901      MRNDLFDLVDYAVQLGVPTSLAPAVSPNLSPETLKAIREHGVKAISISLDGAREETHDEI  136
Pcal_1716      MRADLFELVDYANSLGVPVSLAPAVSKSLDDEALRRIKSSGVKSISISLDGATAETHDEL  136
PAE0596        MRSDLFELIDYANSLSVPVSLAPAVSPNLNTETLKLIKESGVKSISISLDGARPETHDEI  136
Pars_2255      MRADLFELVDYANSLSVPVSLAPAVSPNLTPEVMKEMKQAGVKSVSISLDGAFPETHDEL  136
Msed_0512      KRDDIFEILEYSSAK-ITTALSPSGSR-INVEVAKRIKDTGVSMVSISVDGP-EEIHDEF  120
AF_2413        MRDDVTEIISHAAEKGFRVAIAFSGTEKATEEKLRELKEAGVARVAVSIDGSDEEKHDSF  124
NP_1546A       KRDDLVELVRYGTKQGLRMTLTPSGTEALTPDNIAALVDAGLQRMALSVDAP-ATAHDRF  138
VNG_1185G      ARPDTVELVEHGTDCGLRMTVTPSGTASLTPTAIEALADAGVAQFAVSIDGATPTTHDEF  130
Hlac_1215      ARDDLLELVSYGDDQGLRMTITPSGTQSLTADRIEDLADAGIRRMALSLDGATRESHDRF  131
rrnAC3489      ARGDLPELVEYGTEQGLRMTLTPSGTNSITPERLAELDDAGLRRLALSIDGGDSDAHDTF  131
Hmuk_1679      VRDDVTELVEYGTRQGLGMTLTPSGTESLTPERIEALQDAGLRRMALSLDGGDADSHDAF  130
LRZ99_07755    MRPDIIELVAHAKSKGLRPVFGTNGTL-ITLEMAKRLKTAGALAIGISLDSVDVAKHDRF  117
Memar_0879     LRDDIFEIAEYGTQRGLRMAIGTNGTL-IDDRTAVRLAGAGVRKAAISLDSADPGVHDRF  131
Mboo_0958      MRDDLCTIARYGTDRGLRMVMGTSGYF-LDRPMAARLKEAGIRAAAISLDSADPAVHDSF  126
Mpal_2626      LREDMYDIARYGTEQGLRMVMGTSGYL-IDQETAAKLKEAGIRAVAISLDSKDPATHDAF  149
Mthe_1135      LRQDVFEVARYAAGSGVRVSLASNGTL-ITPEIVDRILLSGISRVSISLDGASAKTNDAT  118
C0624_00890    LRKDIWEIAQYGTDKGLRMCMATNGTL-ITDEICQKMKEVDLKMVSLSLDGSTAAIHDDF  128
Dace_3005      MRPDIFEIAEYGTSKGLRMCMATNGTL-ITDEVCAKMNKADIKMVSLSLDGSTAEIHDDF  128
CSA32_02305    LREDVFDIAAYGTNLGLRMCLATNGTL-VTEDTCRLIKESGIKMVSLSIDGATAAVHDNF  124
DSY57_04415    LREDIFDIATYGAGLGLRMCMATNGTL-VNQEVCRGMKESGIKMVSLSLDGASAAIHDDF  124
B5M56_04105    LRPDIFDIAAHGTSNGLKMVMAPNGTL-ITEESAKKMADAGISRISISIDGATREDHDSF  135
DSCW_66730     LRADIFDIAKYGDNLGLRMVMAPNGTL-ITPQIAEKMAASGIRRISASIDGATKEFHDKF  142
DEB50_09370    LRDDIFDIAAYGDKIGLRMVMAPNGTL-LNEDNVKRLIKSGIKRISVSLDGSTAASHDAF  142
CSA25_02885    LRDDIFDIAAYGDKIGLRMVMAPNGTL-LDEESVTRLMKSGIKRISVSLDGATAASHDAF  142
DVU_0855       MRGDVYELIAYATDKGLRCVMSPNGTL-ITPEHAQRMKASGVQRCSISIDGPDAASHDAF  138
Ddes_0287      IRPDVYELVAYAHSKGLPCAFSPNGTL-ITPETAQKIKNAGVNRCSISIDGADAASHDSF  176
Mbur_1233      VRKDVYEIARYATDKGLRVALATNGTL-LNDGVVKKLKDAGVQRVSISIDGSTAQTHDDF  123
Mbar_A1458     TRPDVFEIARYGTDAGLRVVLATNGTL-LTPEIVEKLRAAGVQRLSVSIDGANAETHDNF  126
MA_0573        TRSDVFEIARYATDAGLRVVLATNGTL-LTPELVEKLRAAGVQRLSVSIDGATAKTHDEF  126
MM_1737        TRTDVFEIARYGTDAGLRVVLATNGTL-LTPEIVKKLRDAGVQRLSISLDGATAKTHDEF  126
                * *   :  :.    .   .                        . *:*.     ::
```

continued on next page

```
Igni_0397     RGINGIFERTVELMNFVKKDVGMFLQINTAVMRDNVEELPEVFKLITDAGVDAWEVFYLI  180
APE_1655      RGVPGTFKASVNIVREA-LAAGLPVQINTTVMKPTVDSLPETLKLLLDLGVDVWEVFYVV  191
Hbut_0035     RGTSGVFEKTLWAIKTF-QEYGVRVQVNTAVMRDNVHDLADIAALLLKLGVKVWEVFYLV  202
ST0127        RGF-GNYRYAINGIKLG-LKYGIQVQVNTLVWKRSYEELPYIAKLLKDLGVKVWEVFFLI  183
M1425_1048    RGL-GSYKYALRGIELG-LKYGIQVQVNTLVWKKSYSELPFVVKLLKEMGVKIWEVFFLI  183
SSO1631       RGL-GSYKYALRGIELG-LKYGIQVQVNTLVWKKSYSELPFVVKLLKEMGVKIWEVFFLI  183
SSO1840       RGL-GSYNYALRGIELG-LKYGIQVQVNTLVWKKSYSELPFLVKLLKELGVKVWEVFFLI  183
Cmaq_1900     RNRNGVFELTLKAISSL-LKAGVKVQVNTTFMKLNVHELPLIVKVLKDLGVYTWEVFFLI  188
Pisl_0113     RGVAGSFRDTVAVIKTA-VDMGIQVQVNTVVWRKSLTELPEVAKLITDLGVRTWEVFFLI  195
Tneu_1901     RGVPGSFRNTLAAIKAA-VDAGVQVQVNTVVWRKSLPELPEVVKLITDLGVKTWEVFFLI  195
Pcal_1716     RGVPGSFAETVSAIKRA-LDLGISVQVNTVVWKKSLSELPDVAYLLRRLGVKVWEVFFLI  195
PAE0596       RGVPGSYKETVNAIKTA-VELGVSVQVNTVVWRKSLAELPEVAYLLKNLGVKVWEVFFLI  195
Pars_2255     RGVPGSYKETVTAIKTA-VEIGLPVQVNTVVWKKSLGELPDVAYLLKNLGVKIWEVFFLI  195
Msed_0512     RGVRGAFKMAKQAVDSL-HEVKLPVQINSTISRYNVDHLQELRKTVEALRPVYWDVFMLI  179
AF_2413       RGVRGTFRMSMMAIENA-KKAGLPFQINTTVTRENIEDLPNIARLCLELGAVMWDVFFVV  183
NP_1546A      RGEAGSFERTREAAEAA-RDAGIPLQTNTTVCATTVETLPAVREFVAEVGAVLWSVFFLV  197
VNG_1185G     RGEAGSFERTLRAARAI-RELGVPLQVNTTVCADTVEALPAIRDLVAELGVALWSVFFLV  189
Hlac_1215     RGEE-SFESTLEAAEAA-SEAGLPLQINTTVCAETVDELPAIRDRVRDLGAVLWSVFFLV  189
rrnAC3489     RGESGSFEATMAAAEAA-RNLDIPLQINTTVCAETVEQLPAIRDLVADLDAVLWSVFFLV  190
Hmuk_1679     RGESGSFEATLAAAEAA-RETDLPLQINTTVCAETVDQLPAIRELVADLGAVMWSVFFLV  189
LRZ99_07755   RATPGAWQGAVQGMRNC-RQAGLPFQIHTTVVDWNYAEVETLTDFAIKEGALAHHTFFLV  176
Memar_0879    RGVAGAWERAVAGIEAC-RDAGIPVQVHTTVTLQNHRDLEGIAEFGESLGVRDFQFFFLV  190
Mboo_0958     RGVSGAWERAVAAIKNC-TEEGIGVQINMTAVRPAAGDIESVVALGKNLGVRDYQVFFPV  185
Mpal_2626     RGLDGVWEKATKAIGHC-HDAGIAVQINMSVMRSAISEVEDLIGLGTSLGVHDYQLFFPI  208
Mthe_1135     RG-EGSFDLALRGIRAL-S-GRVEFQINMTITPANIDELDPILDLAEREGAAAAHIFFMV  175
C0624_00890   RSSPGAFEGTIRGAETL-KRNGIKFLVNSSFTKRNQDDIGATFKLAKGLGATAWYMFMIV  187
Dace_3005     RQCPGAFEGVKRAAETL-TRNGIKFLINSSFTKRNQHDIANTFKLAKSLGATAWYMFMIV  187
CSA32_02305   RNQPGAFTGTMHAIELF-NTHQIDFLVNSSFTRRNKEEAPKIYEMVKTLGATAWYLFMIV  183
DSY57_04415   RTQPGAFDGTMNAIKLF-NENNIPFLINSSFTRRNKKEAPKIYQLVKELGATAWYLFMIV  183
B5M56_04105   RGVDGAYEGALRGAELA-KEAGIDFQINTTVTRLNMEQLPRIMALAESIGAVAHHIFLLV  194
DSCW_66730    RGVDGAFDAAIRGIEYV-KAAGIEFQINTTITKTNLDQIPKILELAEELGAAAHHIFLLV  201
DEB50_09370   RGLDGAFDRAVNGIKTA-KAAGLEFQINTVITKTNLNEIPAILALAESLGAAAHHIFLLV  201
CSA25_02885   RGVDGAFDRAINGIKIA-KAAGLDFQINTVITKTNLDEISAILTLAESLGAAAHHIFLLV  201
DVU_0855      RGVPGAFEQSMRGIGYL-RDAGIEFQINTTVTRDNLHSFKDIFKLCERIGAVAWHIFLLV  197
Ddes_0287     RGVPGAFEASMRGIEYL-KAAGVPFQINTTVTRNNLTSFKKIFELCERIGAAAWHIFLLV  235
Mbur_1233     RGVPGAFESSMRGIEYL-KAGGLGFQINTTITKRNIDEIPAILEIATNIGAEAHHIFLLV  182
Mbar_A1458    RGMPGAFERTLAGIEVL-RKADFPFQINTTVSKRNLEEITKTFELAKELGAVAYHVFFLV  185
MA_0573       RGVPGAFDRTLAGIEVL-RKDGFPFQINTTISRRNLEEIPKTFELAKDLGAVAYHVFFLV  185
MM_1737       RGVPGAFERTLAGIEIL-RKADFPFQINTTISRRNLEEIPKTFELAKELGAVAYHVFFLV  185
              *     :               . .  :                            *  :
```

continued on next page

```
Igni_0397      LTGRASKDLD--LTKDEYWDVSNFLYDASKY-GKTTIRTTEGPFFRVVYRLRTVLDEMGK  237
APE_1655       PTGRAARILD--LTPSEWEDVSNYLYDASRY-GV-LVRTVEGPMFRRIALTRRLLENMGL  247
Hbut_0035      PVGRAQLELN--LTPEEWEDVSHFLYEASKY-GL-VVRTSEGPMFRRVAITRMLLELAGK  258
ST0127         PVGRGTLELD--IPREKYKDVIDFLVEVSRY-NI-VVRTVEAPFFRRAKLEYKEV-----  234
M1425_1048     PVGRGTTELD--IPRDKYKDVIDFLVEATRY-DL-VVRTVEAPFFRRAKLEYTPATT---  236
SSO1631        PVGRGTTELD--IPRDKYKDVIDFLVEATRY-DL-IVRTVEAPFFRRAKLEYTPATT---  236
SSO1840        PVGRGVIELD--IPKEKYKDVIDFLVETTRY-DL-VVRTVEAPFFRRAKLEYTPSTI---  236
Cmaq_1900      HVGRGIELEA--LTPEETEDVVNVLYDVSKY-GF-TVRTVEAPFYRRVVLHRYAFENNEI  244
Pisl_0113      VAGRAKEELD--ITPEEYEAAVQFLVDVSTY-GL-QVRTVEAPFYRRAKLERLEG-----  246
Tneu_1901      VTGRAREELD--ISPEEYEAAVQFLVDVSTY-GI-QVRTVEAPFYRRAKLERLEG-----  246
Pcal_1716      VTGRAKEELD--ISPAEYESAVQFLVDVSTY-GF-QVRTVEAPFYRRAKLERLRG-----  246
PAE0596        VTGRAKEELD--ITPEEYEAAVQFLVDVSTY-GF-QVRTVEAPFYRRAKLERLKG-----  246
Pars_2255      VTGRAREELD--ITPEEYEAAVQFLVDVSTY-GF-QVRTVEAPFYRRAKLERLEG-----  246
Msed_0512      PTGRATKEMM--ITSEQAEEVMRTITKW-RM-EGLNVRMTCAPYLVRVMNEMGVVR----  231
AF_2413        PTGRAKAEMM--PTPQQFEDVLCWLYDLSKK-TGLNVKSSAATHLRRIELMRDRGE----  236
NP_1546A       PVGRGQILDP--IQPERAESVLEWLQDVSEA-APFGVKTTEAPHYRRVALQRNGSA----  250
VNG_1185G      PVGRGRALDP--VSPARAEEVMAWLDGVARS-EAFGVKTTEAPMYRRVRAQRGGDG----  242
Hlac_1215      AVGRGRILEP--IAPERAEDVMEWLHGVAES-EPFGVKTTEAPFYRRVGLQSDGD-----  241
rrnAC3489      PVGRGRVLTP--IDPERAERVLKWLHEVSDE-ASFGLKTTEAPHYRRVAMEQQDEG----  243
Hmuk_1679      PVGRGRVLTQ--ISPDRAERVMEWLHEVSES-EPFGLKTTEAPHYRRVTIERERDG----  242
LRZ99_07755    PTGRAVNIEQETLKAEQYEKLLHRIMTKQQQ-VAIELKPTCAPQFMRIAKQLGVK-----  230
Memar_0879     PTGRGKEVVD--ISPEMYESLIRRILRLRAD-RGLSIRPTCAPQYVRIAAGMGLP-----  242
Mboo_0958      PTGRAGGTGP--ENPREYEDVIRRVLLKYCD-SNVNLRPTCAPQFRRIAADLGVT-----  237
Mpal_2626      PTGRARQIEP--RSPEEYEEMIRRILIRYRD-SRINIRPTCAPQFRRIADECGIA-----  260
Mthe_1135      PTGRGRAV--ECISPEMQRSLLE---RIASEERSIEIRPTCAPQYGRVLMEKKGS-----  225
C0624_00890    PTGRGEEIMNELITKEDYEEILSWHYEQEKNEDDILMRPTCAPHYYRIVPQMAKAEGVDF  247
Dace_3005      PTGRGEEIMNELVSKEDYEEILSWHYEQEKNEDDILMRPTCAPHYYRIVPQMAKAEGVDF  247
CSA32_02305    PTGRGEDIMAELIPEEEYEDILNWHYDMETEESDLLVRPTCAPHYYRIVLQRSKEEGSRF  243
DSY57_04415    PTGRGEEIMEELIPESEYEDMLNWHYDMEKKESDMLVRPTCAPQYYRIVLQRAKKEGEKF  243
B5M56_04105    PTGRGREMAEQAISAQDYENTLNWFYDQQKI-SSLQLKATCAPQYYRVLRQRARMENISV  253
DSCW_66730     PTGRGKYIVDQAIDAESYESTLNWFYDQREK-TSLQLKATCAPHYYRILRQRARDEGKTI  260
DEB50_09370    PTGRGKYIVDTAIDAKEYEETLNWFYDQRDK-TSLQLKATCAPHYYRILRQRAKADGKKV  260
CSA25_02885    PTGRGKYIVDTAIDAKEYEETLNWFYDQRDK-TSLQLKATCAPHYYRILRQRAKAEGRKV  260
DVU_0855       PTGRAAGLSDQVISAAEYEEVLNWFYDFRKT-TSMHLKATCAPHYYRIMRQRAKEEGVSV  256
Ddes_0287      PMGRAAGLADQVITAQEYEDVLHWLYDFRKT-TKMHLKATCAPHYYRIMRQRAKEEGVSV  294
Mbur_1233      PTGRGKELENEEIPPAEYERVLNWFYDQQKH-VKIQLKATCAPHYFRIMRQRAKREGTEV  241
Mbar_A1458     PTGRGDE--SDEVSPADYERILHWFYEMQKE-SKIQLKATCAPHYFRIMRQQAKKEGIEI  242
MA_0573        PTGRGEE--SDEVSPADYERVLHWFYDMQKE-SEIQLKATCAPHYFRIMRQRAKKEGIEI  242
MM_1737        PTGRGEE--SDEVSPADYERVLHWFYDMQKE-SEIQLKATCAPHYFRIMRQRAKKEGIEI  242
                 **.                               ::   .
```

continued on next page

```
Igni_0397     GPE-ELGVGELYYKLRERLEELMGPVP--------HGPPKQKRAPSAYTRDGYGIIFVAY   288
APE_1655      DWRNRLRPGSLYHKLARKTLELLG--D----------PPGEARAQTTGTRDGKGVIFVSN   295
Hbut_0035     NPDEALNTGPLYRQLVSRLRQLLG--E----------PQGKPLASTTGTRDGKGVIFVSY   306
ST0127        -----------KNELIRKLKELLG--E----------SKSPVDKSILPTRDGAGVIFISY   271
M1425_1048    ----------NDNELVSTLRELLG--E----------PVKEADKSILPTRDGAGVIFIGY   274
SSO1631       ----------NDNELVSTLRELLG--E----------PVKEADKSILPTRDGAGVIFIGY   274
SSO1840       ----------KSNELVSKLRELLG--E----------PVKDVDKSVLPTRDGSGVIFIGY   274
Cmaq_1900     N--IKPNLGPLYRQLYEGLIKVMGNEP----------PKLTKRPMVARTRDGDGIIFVAY   292
Pisl_0113     ----KTYDSPLYRKLVGRLRELMG--P----------PRRGIDPTIVPTRDGFGIIFVAY   290
Tneu_1901     ----KTYDSPLYRKLVERLGELMG--P----------PRRGVDPTVVPTRDGFGIIFVAY   290
Pcal_1716     ----KAYSDLLYDKLVARLRELLG--P----------PQRGVDPTIVPTRDGFGIIFVGH   290
PAE0596       ----REFGHPLYNQLVERLRNLMG--P----------PVRAVDPTIVPTRDGFGIIFIAY   290
Pars_2255     ----RIYDHPLYLQLVDKLRKLLG--P----------PTRGVDPTIVPTRDGFGIIFVAY   290
Msed_0512     ----P----------------LP----------PDKNYGRRSVNGARGCMAGNGYAFVAY   261
AF_2413       ----MPAVGELYYRLLERIEDFPEGEGIVVAGGHGKSLSTDGIRRAIGITDGRGMFFISH   292
NP_1546A      ----T------------------------------DAPDADAIGRRGGIIAGDGFAFVSH   276
VNG_1185G     -----------------------------------------APQRRAGVTAGDGFAFVSH   261
Hlac_1215     ---------------------------------------EEATRRRGGITAGRGFAFVSH   262
rrnAC3489     ----A-----------------------------------SGLKRRMGIRAGKGFAFVSH   264
Hmuk_1679     ----E-------------------------------AGENDGLQRRTGIRAGQGFAFVSH   267
LRZ99_07755   -----------------------------------------M-RFSKGCLAGTGYCIISP   248
Memar_0879    -----------------------------------------VAEGERGCIAGIRYCRIDP   261
Mboo_0958     -----------------------------------------KPDWGRGCIAGISYCRIYA   256
Mpal_2626     -----------------------------------------NPAWGRGCLAGITYCRIFA   279
Mthe_1135     -----------------------------------------QARTAGGCIAGIRFVFISR   244
C0624_00890   ERRSL----------------------------------TFSTGGGKGCIAAQTICLIDC   273
Dace_3005     KRRSL----------------------------------TFSTGGGKGCIAAQTICLIDC   273
CSA32_02305   RRRSL----------------------------------KFSTGGSKGCLAGQLICLIDV   269
DSY57_04415   KRRNL----------------------------------KFSTGGSKGCLAGQLICLIDV   269
B5M56_04105   SFKTH----------------------------------GLD-AVTRGCLGGVSFCFISH   278
DSCW_66730    SFESH----------------------------------GLD-AVTRGCLAGTGFCFISH   285
DEB50_09370   SFETH----------------------------------GLD-AVTRGCLAGTGFCFISH   285
CSA25_02885   SFKTH----------------------------------GLD-AVTRGCLAGTGFCFISH   285
DVU_0855      TPDNF----------------------------------GMD-AMTRGCLGGTGFCFISH   281
Ddes_0287     TPENF----------------------------------GMD-ALTRGCLGGTGFCFISH   319
Mbur_1233     TVKTH----------------------------------GYE-AMTRGCLGGISFCFVSS   266
Mbar_A1458    SVKTH----------------------------------GYE-AMTKGCLGGTGFCFVSS   267
MA_0573       SVKTH----------------------------------GYE-AMTKGCLGGTGFCFVSS   267
MM_1737       SVKTH----------------------------------GYE-AMTKGCLGGTGFCFVSS   267
                                                                 .     :
```

continued on next page

```
Igni_0397      NGDVYPSGFLPYKVGNVREASLKEIYQNSKALKMIRDPSNFRPPCGTCKFNFMCGGSRAR   348
APE_1655       KGLVYPSGFLPYPVGDVRKSSLKEIYQSSPELEGLRK-AVFKGRCGRCEFSQLCGGSRAR   354
Hbut_0035      NGTVYPSGFMPYPLGNIRVKSLVEIYRENLILKRLRG-ARFEGRCGRCEFREICGGSRAR   365
ST0127         NGDIYPSGFLPLKLGNVREDRLIDVYRNSELLKMIKA-GKLKGKCGICAFSNICGGSRAR   330
M1425_1048     NGDVYPSGFLPLYLGNVKKESLVDIYRKSEVLKKIKD-SRFEGKCGICKYNNICGGSRAR   333
SSO1631        NGDVYPSGFLPLYLGNVKKESLVDIYRKSEVLKKIKD-SRFEGKCGICKYNNICGGSRAR   333
SSO1840        NGDVYPSGFLPLYLGNVRKESIVDIYRKSEVLKIIKD-GRFDGKCGVCKFNNICGGSRAR   333
Cmaq_1900      NGDVSPSGFLPIKLGNVKEESLVKIYRENPVLLRIRR-GEYGGRCGLCEFRFICGGSRAR   351
Pisl_0113      DGTVYPSGFLPYPLGNVRRRSLVEIYREHPLLQKMRR-GEFGGRCGVCKYKDICGGSRAR   349
Tneu_1901      DGTVYPSGFLPYPLGNVRRRSLVEIYRDHPLLQKMRR-GEFGGRCGVCKYKDICGGSRAR   349
Pcal_1716      DGTVTPSGFLPYPLGNVRKRRLVDIYRNHPLLVKMRR-GEFEGRCGVCEYKDICGGSRAR   349
PAE0596        DGTVHPSGFLPYPLGNVRKQSLVKIYREHPLLLKMRR-GEFGGRCGVCEYKDICGGSRAR   349
Pars_2255      DGTVHPSGFLPYPLGNVRRQSLVEIYRNHSLLQKMRR-GEFGGRCGVCRYKDICGGSRAR   349
Msed_0512      DGTVYPCGFLPIPAGNVRFRRFSEIYEQSPVFKSLREPSKLGGKCGLCEYRSVCGGCRAR   321
AF_2413        IGEVYPSGFLPIVAGNVRNTSLKEIYYSSEIFVNLRDPDRLKGKCGRCEYRKICGGSRAR   352
NP_1546A       TGELFPSGFLPKSAGNVTDSDLVSLYQDSTLFESLRDRDRLKGKCGACEFRHVCGGSRSR   336
VNG_1185G      TGAVYPSGFLPSSAGNVRERSVVDSYRNSELFQRLRDTEALTGKCGACSFRTVCGGSRSR   321
Hlac_1215      TGEAYPSGFLPESAGNVHDRSIVDIYRNGDLFESLREPDRLKGKCGACEFRQVCGGSRSR   322
rrnAC3489      TGEVFPSGFLPKSAGSVREESVVDIYRDSPLFQQLRDDDALTGKCGACRYRTVCGGSRSR   324
Hmuk_1679      TGEMYPSGFLPESAGNVRSEGVVDLYRESALFQRLRDDSALEGKCGACPYRGVCGGSRSR   327
LRZ99_07755    KGIVQPCAYLNIPAGNVRETPFSEIWRDNAVFNRLRNQ-PLQGGCGTCDYQKICGGCRAR   307
Memar_0879     TGEVTPCPYLPLGLGNIRRTPFAEIWNGSEVFAALRSGEGLRGKCGACEYRSACGGCRAR   321
Mboo_0958      NGDVTPCPYLPVSAGNVRDIPFDRIWNESHLFHALRDPNRLTGKCGRCEYKTTCGGCRAR   316
Mpal_2626      NGDVTPCPYLPVSAGNVRTTPFSEIWNNSPLFAALRDPSRLTGKCGRCSFKTSCGGCRAR   339
Mthe_1135      TGDVFPCGYFPLSAGSIRDRSFSEIWSSSPLLNDLRER-RLKGRCGSCNYVRICGGCRAR   303
C0624_00890    FGNLKPCSYFHSSVGNVKQIPFKELWFNNKVFNDLRDFSKYKGKCGECEFLNVCGGCRAR   333
Dace_3005      FGNLKPCSYFHSSVGNVKQIPFKDLWFNSKVFNDLRDFSKYKGKCGECEFINVCGGCRAR   333
CSA32_02305    DGNILPCSYFPLSDSNIRDKSFKDIWENSPLLTDMRNFSQYKGSCGRCEYIQVCGGCRAR   329
DSY57_04415    DGNVLPCSYFPKAAGNIRSQSFQDIWENSTLFHELRDFKSYKGSCGHCEYVNVCGGCRAR   329
B5M56_04105    VGIVQPCGFLELNCGDVTSQHFGEIWRDSVIFNNLRNYDMLGGKCGICEYKRVCGGCRAR   338
DSCW_66730     TGIVQPCGYTDVKCGDITRDTFGHVWRNSPVFLKLRDFKQLEGKCGRCEYRAVCGGCRAR   345
DEB50_09370    VGRVQTCGFLDVTCGDIKTSHFKDVWENSEVFNKLRDFNNLEPKCGICEYKQVCGGCRAR   345
CSA25_02885    VGRVQTCGFLDVTCGDIKTHHFKDVWENSDVFNKLRNFNNLDPKCGLCEYKQVCGGCRAR   345
DVU_0855       TGQVQPCGYLELDCGNVRNTPFPEIWRKSEHFRQFRTQEEYTGKCGPCEYHKVCGGCRAR   341
Ddes_0287      VGQVQPCGYLELDCGNVRQTPFPKIWRESKHFLQFRDQSCYSGKCGECEYHKVCGGCRAR   379
Mbur_1233      TGDVQPCGYLPVIAGNIKEKSFKEIWEDSVLFNDLRDYDKLGGKCGRCSYKNVCGGCRAR   326
Mbar_A1458     VGKVFPCGYLPVLAGNIREQPFREIWENAEVFRKLRDPEELKGKCGICEYKKVCAGCRAR   327
MA_0573        VGEVYPCGYLPVLAGNIREQPFKEIWENSEVFRKLRDPEELKGKCGICEYKKVCAGCRAR   327
MM_1737        VGEVYPCGYLPVLAGDIRAQPFKDVWENSEVFRKLRDPEELKGKCGICEYKKVCAGCRAR   327
                *    . :     ..:    .   :     :  ::        ** * :   *.*.*:*
```

continued on next page

```
Igni_0397       AYSY------------------FKDPFGHDPACKLPEVLESL-DPQVVEEALRPFGRAKX_5  393
APE_1655        AYSY------------------TGDPLGEDPACAYRPGEFRSLLQELGVGEADVYGLVEX_8  403
Hbut_0035       AYAV------------------TGKPFGEDPACPYRPGTFSVMVSKLGVRVENVVREIEX_9  415
ST0127          AYAV------------------YGDPLAEDPACPY-------------------------  347
M1425_1048      AFAV------------------YNNPFAEDPMCPY-------------------------  350
SSO1631         AFAV------------------YNNPFAEDPMCPY-------------------------  350
SSO1840         AFAV------------------YNNPFAEDPMCPY-------------------------  350
Cmaq_1900       AYAE------------------YGDPLAEDPACVYSPGTIRLPESTLSP-----------  382
Pisl_0113       AFAY------------------FKDPLAEDPACIYSPPL---------------------  370
Tneu_1901       AFAY------------------FKDPLAEDPACVYKP-----------------------  368
Pcal_1716       AFAV------------------FKNPLAEDPACVYIPSSWKSVEMTR-------------  378
PAE0596         AFAY------------------FKDPLAEDPACVYKPHRNL-------------------  372
Pars_2255       AFAY------------------YKDPLAEDPACIYKPT----------------------  369
Msed_0512       AFSL------------------TENFMDEDPFCTYVPRTLRVRA----------------  347
AF_2413         AYAV------------------HGDYLAEEPCCIYIPQSSR-------------------  375
NP_1546A        AYAV------------------TGDPLASDPLCAYRPSGYDGPLPDRQAAADR-------  371
VNG_1185G       AYAT------------------TGEPMASDPLCPHVPDGYDGPLPDRQATADD-------  356
Hlac_1215       AFAT------------------TGDPLESDPLCPYVPDGYDGELPPTLKDGFEGSTSAPX_4  367
rrnAC3489       AYAT------------------TGDPLAADPLCDYRPDGFEGSVPDQHPAD---------  357
Hmuk_1679       AYAA------------------TGDPMGSDPLCSFVPDEYDGPLPATHGERVPTD-----  364
LRZ99_07755     AYYY------------------HGDYMAEEPWCLYHGRKGY-------------------  330
Memar_0879      AYGVTQE------------RSGTGDCLAEDPWCLYEPGVR--------------------  349
Mboo_0958       AYRGAEAFSSRWCDGLLKPSAIAGELCAEDPWCPYEPGGS--------------------  356
Mpal_2626       AYRQEDAASPLWCDGLATPDIVNGEICGEDPWCPYQPPDVIP------------------  381
Mthe_1135       AYAL------------------TGDYLGEDPTCAWRGSIG--------------------  325
C0624_00890     ADAV------------------YGDYMQQEPFCNYVPERMRKKMHKEAEEMAPK------  369
Dace_3005       ADAV------------------YGDYMAEEPFCNYIPRKTRLRMEKEAAENRGE------  369
CSA32_02305     AYAM------------------TGDYMAEEPFCTYQPSRKMRTQL---------------  356
DSY57_04415     AYAV------------------TGDYLAPEPFCSYQPK----------------------  349
B5M56_04105     AYEA------------------TGDYLAEEPLCVYQPAKQSQPL----------------  364
DSCW_66730      AYEA------------------TGNYMAEEPLCAYQPEKAAPC-----------------  370
DEB50_09370     AYEA------------------TGNYLAQEPLCTYQPPRH--------------------  367
CSA25_02885     AYEA------------------TGNYLAQEPLCTYQPARHKSAV----------------  371
DVU_0855        AYNM------------------SGDHMAEEPLCSYKPRRMTPCR----------------  367
Ddes_0287       AHSM------------------DGDHMGEEPLCTYIPAKMRKKGRDGKSGEEK-------  414
Mbur_1233       AYAA------------------TGDYMAEEPYCIYNPR----------------------  346
Mbar_A1458      AYAA------------------TGDYLEEEPYCIYRPGKK--------------------  349
MA_0573         AYAA------------------TGDYLEEEPYCIYRPGKK--------------------  349
MM_1737         AYAA------------------TGDYLEEEPYCIYRPGKK--------------------  349
                *                       .    :* *
```

Figure S1. Amino acid sequence alignment of AhbD sequences from different archaea and sulfate-reducing bacteria. Conserved N-terminal cysteine residues coordinating the RS cluster are highlighted in violet purple, C-terminal cysteine residues in raspberry, and partially conserved cysteine residues of the full SPASM motif in salmon. Sequences are specified by gene numbers.

Figure S2

```
anSME_CPE0635      6  LLIKPASSGCNLKCTYCFYHSLSDNRNVKSYGIMRDEVLESMVKRVLNE--ADGHCSFAF   63
SuiB_STRSU       108  ELVIYPSMYCDLKCGFCFLANREDRN----------AKPAKDWERILRQAKDNGVLSVSI  157
PQQE_KLEPN        13  WLLAELTYRCPLQCPYCSNPLDFARQDK--------ELTTEQWIEVFRQARAMGSVQLGF   64
MFTC_MYCTU        21  CLTWELTYACNLACVHCLSSSG-KRDPG--------ELSTRQCKDIIDELERMQVFYVNI   71
DVU_0855          22  LIAWEVTRSCNLACKHCRAEAHMEPYPG--------EFSTDEAKALIDTFPDVGNPIIIF   73
Ddes_0287         60  LIAWEVTRSCNLACKHCRAEAHPEPYPG--------ELSTAEAKALIDTFTEVGKPIIIF  111
Mbur_1233          7  LIAWELTWGCNLACVHCRGSSTSEIPEG--------ELSTSEAKHFVDEIVEMGDPILIL   58
Mbar_A1458        10  LIAWELTAGCNLNCVHCRGASTSSVPAG--------ELTTDEAKHFIDEVASIGKPILIL   61
                       :    :  * * * .*                            ..          . :

anSME_CPE0635         QGGEPILAGLEFFERLMELQRKHNYKNLKIYNSLQTNGTLIDESWAKFLSENKF-LVGLS  122
SuiB_STRSU            LGGEPTRYFD-IDN----LLI--ACEELKIKTTITTNAQLIKKSTVEILAKSKYITPVLS  210
PQQE_KLEPN            SGGEPLTRKD-LPE----LIR--AARDLGFYTNLITSGIGLTESKLDAFSEAGLDHIQIS  117
MFTC_MYCTU            GGGEPTVRPD-FWE----LVD--YATAHHVGVKFSTNGVRITPEVATRLAATDYVDVQIS  124
DVU_0855              TGGDPMMRGD-VYE----LIA--YATDKGLRCVMSPNGTLITPEHAQRMKASGVQRCSIS  126
Ddes_0287             TGGDPMIRPD-VYE----LVA--YAHSKGLPCAFSPNGTLITPETAQKIKNAGVNRCSIS  164
Mbur_1233             TGGEPLVRKD-VYE----IAR--YATDKGLRVALATNGTLLNDGVVKKLKDAGVQRVSIS  111
Mbar_A1458            SGGEPLTRPD-VFE----IAR--YGTDAGLRVVLATNGTLLTPEIVEKLRAAGVQRLSVS  114
                       **:*      . :    :          .   :  ..  :       :         :*

anSME_CPE0635         MDGPKEIHNLNRKDCCGLDTFSKVERAAELFKKYKV-EFNILCVVTSNTARHVNKIYRYF  181
SuiB_STRSU            LQTLDSKLNFELMGVR--PD--RQIKLAKYFNEVGK-KCRINAVYTKQSYEQIIELVDFC  265
PQQE_KLEPN            FQASDEVLNAALAGNK--KAFQQKLAMAKAVKARDY-PMVLNFVLHRHNIDQLDKIIELC  174
MFTC_MYCTU            LDGATAEVNDAIRGT---GSFDMAVRALQNLAAAGFAGVKISVVITRRNVAQLDEFATLA  181
DVU_0855              IDGPDAASHDAFRGVP--GAFEQSMRGIGYLRDAGI-EFQINTTVTRDNLHSFKDIFKLC  183
Ddes_0287             IDGADAASHDSFRGVP--GAFEASMRGIEYLKAAGV-PFQINTTVTRNNLTSFKKIFELC  221
Mbur_1233             IDGSTAQTHDDFRGVP--GAFESSMRGIEYLKAGGL-GFQINTTITKRNIDEIPAILEIA  168
Mbar_A1458            IDGANAETHDNFRGMP--GAFERTLAGIEVLRKADF-PFQINTTVSKRNLEEITKTFELA  171
                      ::      :    .                .         :  .    .   .

anSME_CPE0635         KEKDFKFLQFINCLDPLYEEKGKYNYSLKPQDY-TKFLKNLFDLW------YEDFLNGNR  234
SuiB_STRSU            IENKIDRFSV-ANYSEVTGYTKIKKK-YDLADL-RRLNEYVTD----YITQREANLNFAT  318
PQQE_KLEPN            IELEADDVEL-AT---------------------CQFYGWAFLNREGLLPTREQIARAEQ  212
MFTC_MYCTU            SRYGAT-LRI-TRLRPSGRGTDVWADLHPTADQQVQLYDWLVSKGERV----------L  228
DVU_0855              ERIGAVAWHI-FLLVPTGRAAGLSDQVISAAEY-EEVLNWFYDFRKTTSMHL-----KAT  236
Ddes_0287             ERIGAAAWHI-FLLVPMGRAAGLADQVITAQEY-EDVLHWLYDFRKTTKMHL-----KAT  274
Mbur_1233             TNIGAEAHHI-FLLVPTGRGKELENEEIPPAEY-ERVLNWFYDQQKHVKIQL-----KAT  221
Mbar_A1458            KELGAVAYHV-FFLVPTGRGDE--SDEVSPADY-ERILHWFYEMQKESKIQL-----KAT  222
                       .       .                          .

anSME_CPE0635         VSIRYFDGLL-----ETIL--LG-----KSSSCGMNGTCTCQFVVESDGSVYPCDFYVLD  282
SuiB_STRSU            EGCHLFTAYP-----ELINNSIEFSEFDEMYYGCRAKYTKMEIM--SNGDILPCIAFLGV  371
PQQE_KLEPN            VVADYRQKMAASGN-LTNLLFVTPDYYEERPKGCMGGWGSIFLSVTPEGTALPCHSARQL  271
MFTC_MYCTU            TGDSFFHLAP-----------LGQSGALAGLNMCGAGRVVCLID--PVGDVYACPFAIHD  275
DVU_0855              CAPHYYRIMRQRAKEEGVSVTPDNFGMDAMTRGCLGGTGFCFIS--HTGQVQPCGYLEL-  293
Ddes_0287             CAPHYYRIMRQRAKEEGVSVTPENFGMDALTRGCLGGTGFCFIS--HVGQVQPCGYLEL-  331
Mbur_1233             CAPHYFRIMRQRAKREGTEVTVKTHGYEAMTRGCLGGISFCFVS--STGDVQPCGYLPV-  278
Mbar_A1458            CAPHYFRIMRQQAKKEGIEISVKTHGYEAMTKGCLGGTGFCFVS--SVGKVFPCGYLPV-  279
                                                                .     *    *

anSME_CPE0635         KWRLGN-IQDMTMKELFETNKNHEFIKSSFKVHEECKKCKWFKLCKGGCRRCRDSKEDSD  341
SuiB_STRSU            NQTKQN-AFEKDLLDVWYDDPLYGGIRSFRTKNSKCLSCGLLKICEGGCYVNLIKEKSPE  430
PQQE_KLEPN            PVAFPS-VLEQSLESIWYDSFGFNRYRGYDWMPEPCRSCDEKEKDFGGCRCQAFMLTGSA  330
MFTC_MYCTU            HFLAGNVLSDGGFQNVWKNSSLFRELREPQS-AGACGSCGHYDSCRGGCMAAKFFTGLPL  334
DVU_0855              --DCGN-VRNTPFPEIWRKSEHFRQFRTQEEYTGKCGPCEYHKVC-GGCRARAYNMSGDH  349
Ddes_0287             --DCGN-VRQTPFPKIWRESKHFLQFRDQSCYSGKCGECEYHKVC-GGCRARAHSMDGDH  387
Mbur_1233             --IAGN-IKEKSFKEIWEDSVLFNDLRDYDKLGGKCGRCSYKNVC-GGCRARAYAATGDY  334
Mbar_A1458            --LAGN-IREQPFREIWENAEVFRKLRDPEELKGKCGICEYKKVC-AGCRARAYAATGDY  335
                           .   :  : .::     .   :        *  *   .   .**

anSME_CPE0635         LELNYYCQSYKEFFEYAFPRLI------NVANNIK-------------------------  370
SuiB_STRSU            YFRDSVCQL---------------------------------------------------  439
PQQE_KLEPN            DNADPVCSKSPHHHKILEARRE------AACSDIKVSQ----LQFRNRTRSQLIYQTRDL  380
MFTC_MYCTU            DGPDPECVQGHSEPALARERHLPRPRADHSRGRRVSKPVPLTLSMRPPKRPC--N-ESPV  391
DVU_0855              MAEEPLCSYKPRRMTPC--------R----------------------------------  367
Ddes_0287             MGEEPLCTYIPAKMRKK--------GRDGKSGEEK-------------------------  414
Mbur_1233             MAEEPYCIYNPR------------------------------------------------  346
Mbar_A1458            LEEEPYCIYRPGKK----------------------------------------------  349
                         :  *
```

Figure S2. Amino acid sequence alignment of AhbD with the SPASM domain-containing Radical SAM enzymes anSME, SuiB, PqqE and MftC. Cluster-coordinating cysteine residues are highlighted in color as in Figure S1. Sequences are specified by UniProt entry names or gene numbers.

Figure S3

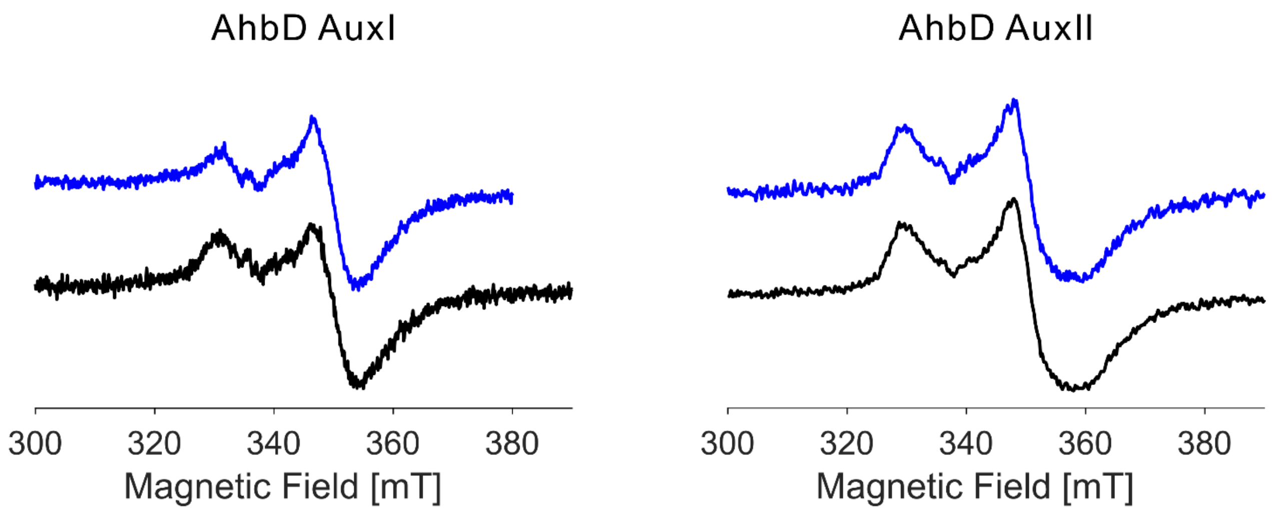

Figure S3. EPR Cw X-band spectra of the dithionite reduced AhbD variants AuxI (left) and AuxII (right) recorded before (blue) or after (black) iron-sulfur cluster reconstitution. Spectra were recorded at T = 13 K and P_{MW} = 1 mW. All other experimental parameters are given in the main text.

Figure S4

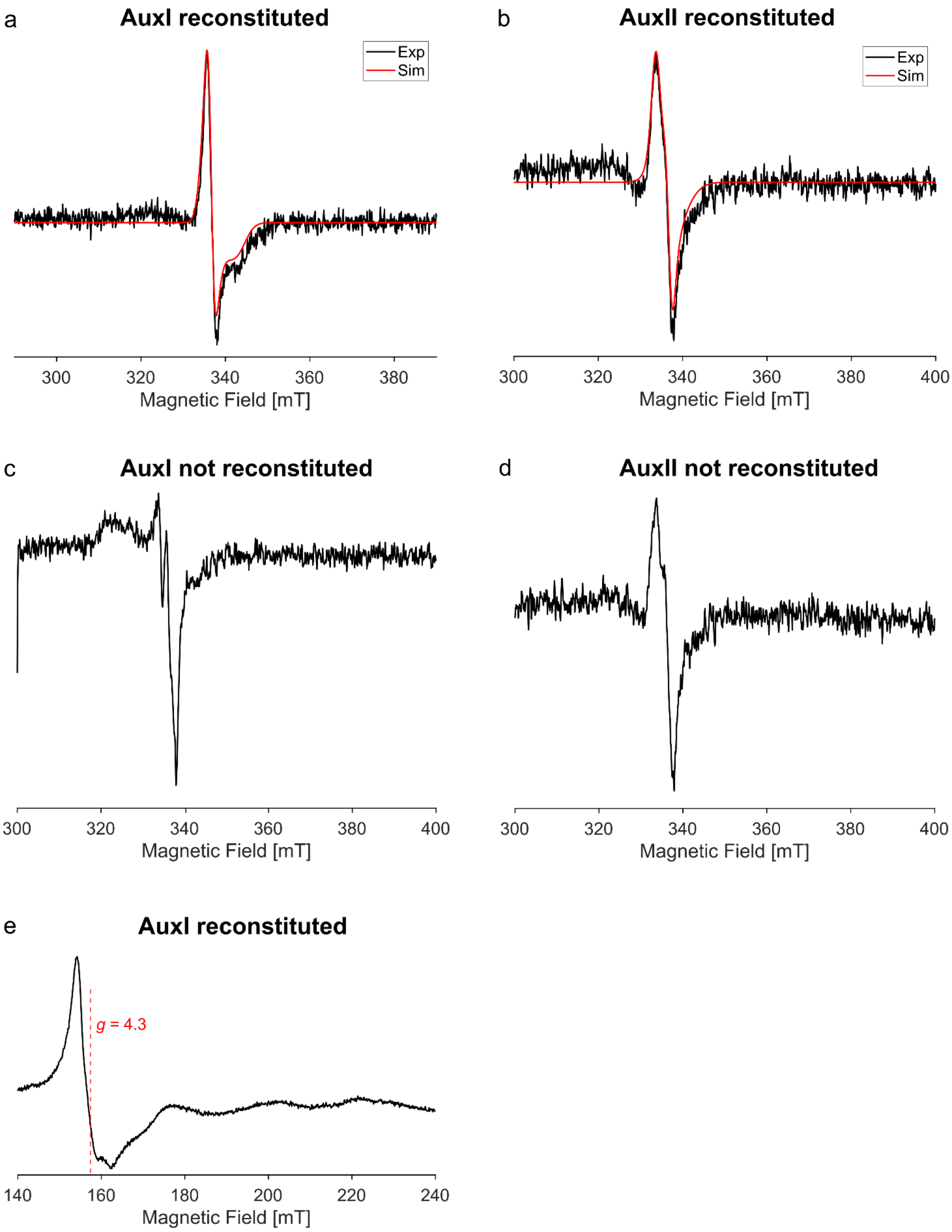

Figure S4. EPR Cw X-band spectra of the oxidized AhbD variants AuxI (a, c) and AuxII (b, d) recorded before (c, d) or after (a, b) iron-sulfur cluster reconstitution. Spectra a and b were simulated (red line) with g_x; g_y; g_z = 2.016; 2.010; 1.973 and g_x; g_y; g_z = 2.026; 2.007; 1.998, respectively. e) shows the signal of 'free' Fe(III) at g = 4.3 contained within the reconstituted AuxI variant. Similar spectra with the same intensity of the peak at g = 4.3 were obtained from the other samples. Spectra were recorded at T = 13 K and P_{MW} = 1 mW. All other experimental parameters are given in the main text.

Figure S5

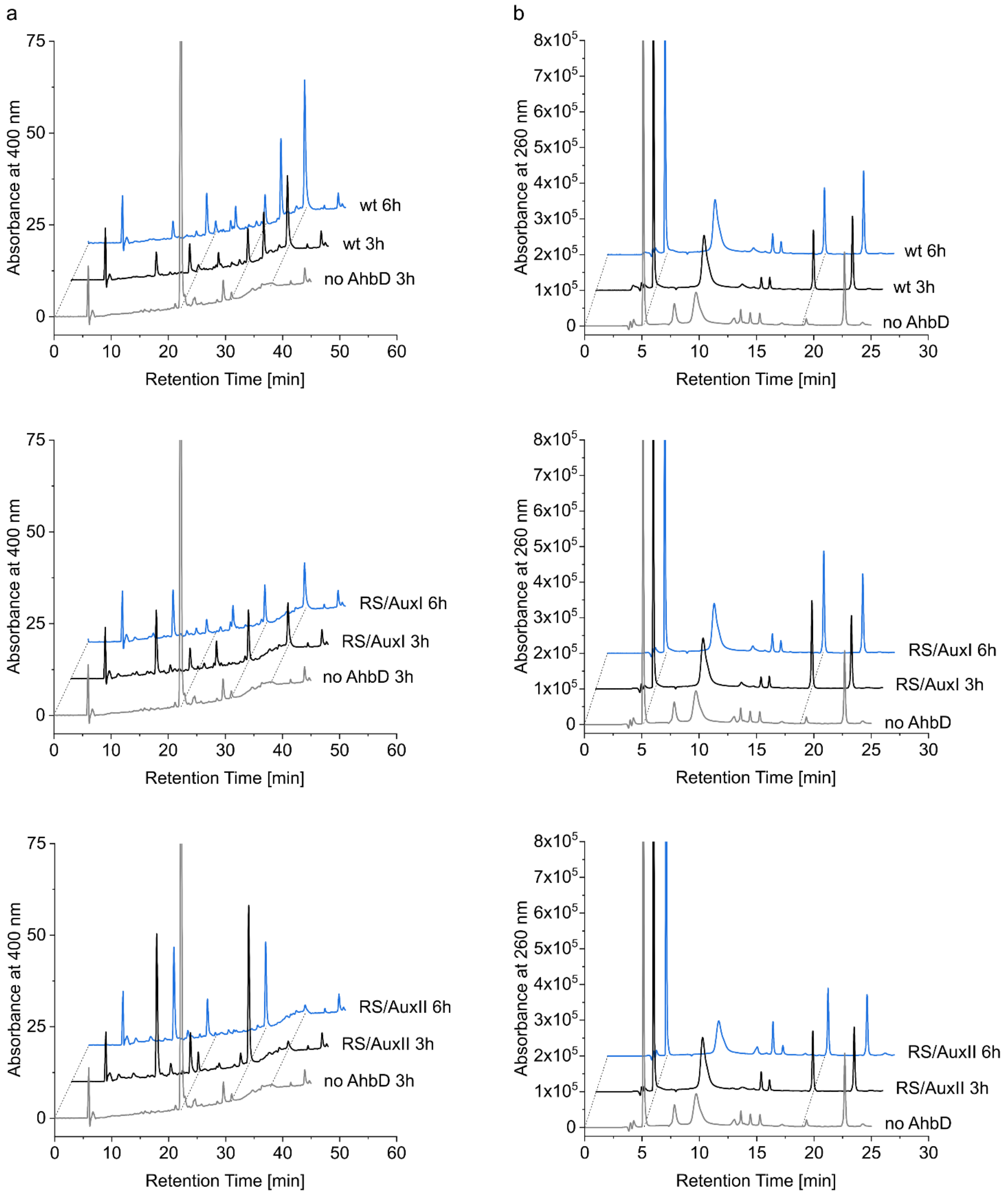

Figure S5. HPLC analysis of enzyme activity assay mixtures containing AhbD wt (top), variant RS/AuxI (middle) or RS/AuxII (bottom). a, HPLC separation of tetrapyrroles extracted from enzyme activity assay mixtures after 3 and 6 hours of incubation. FeCopro elutes at a retention time of 22.1 min, the monovinyl-intermediate at 31 min and heme at 37.8 min. b, HPLC separation of SAM cleavage products formed after 3 and 6 hours of incubation. SAM and 5′-dA elute at retention times of about 5 and 18.9 min, respectively.

Table S1

Table S1. Oligonucleotide primers for site-directed mutagenesis of the *ahbD* gene.

Primer designation	Sequence (5′ → 3′)[1]
Mbar_C23A_C26A_rv	CTTGCACCACGAGCATGAACAGCATTCAGATTACAACC
Mbar_C23A_C26A_fw	GGTTGTAATCTGAATGCTGTTCATGCTCGTGGTGCAAG
Mbar_C256A_rv	AACCGGTGCCACCCAGCGCACCTTTGGTCATTGCTTC
Mbar_C256A_fw	GAAGCAATGACCAAAGGTGCGCTGGGTGGCACCGGTT
Mbar_C274A_rv	GAACCGGCAGATAACCCGCCGGAAAAACTTTACCAACGC
Mbar_C274A_fw	GCGTTGGTAAAGTTTTTCCGGCGGGTTATCTGCCGGTTC
Mbar_C312A_C315A_rv	CTTTTTTATACTCGGCGATGCCAGCTTTACCTTTCAGTTCTTCC
Mbar_C312A_C315A_fw	GGAAGAACTGAAAGGTAAAGCTGGCATCGCCGAGTATAAAAAAG
Mbar_C321A_rv	CACGACAACCTGCCGCAACTTTTTTATACTCGGCGATG
Mbar_C321A_fw	CATCGCCGAGTATAAAAAAGTTGCGGCAGGTTGTCGTG
Mbar_C324A_rv (for Q5 Kit)	ACTTTTTTATACTCGCAGATG
Mbar_C324A_fw (for Q5 Kit)	TTGTGCAGGTGCTCGTGCACGTG

[1] Mutated codons are underlined.
